# A multifaceted architectural framework of the mouse claustrum complex

**DOI:** 10.1002/cne.25539

**Published:** 2023-10-02

**Authors:** Joachim S. Grimstvedt, Andrew M. Shelton, Anna Hoerder‐Suabedissen, David K. Oliver, Christin H. Berndtsson, Stefan Blankvoort, Rajeevkumar R. Nair, Adam M. Packer, Menno P. Witter, Clifford G. Kentros

**Affiliations:** ^1^ Kavli Institute for Systems Neuroscience NTNU Norwegian University of Science and Technology Trondheim Norway; ^2^ Department of Physiology, Anatomy & Genetics University of Oxford Oxford UK; ^3^ Institute of Neuroscience University of Oregon Eugene Oregon USA

**Keywords:** claustrum, cytoarchitecture, delineation, fiber‐architecture, genetic markers, rabies tracing

## Abstract

Accurate anatomical characterizations are necessary to investigate neural circuitry on a fine scale, but for the rodent claustrum complex (CLCX), this has yet to be fully accomplished. The CLCX is generally considered to comprise two major subdivisions, the claustrum (CL) and the dorsal endopiriform nucleus (DEn), but regional boundaries to these areas are debated. To address this, we conducted a multifaceted analysis of fiber‐ and cytoarchitecture, genetic marker expression, and connectivity using mice of both sexes, to create a comprehensive guide for identifying and delineating borders to CLCX, including an online reference atlas. Our data indicated four distinct subregions within CLCX, subdividing both CL and DEn into two. Additionally, we conducted brain‐wide tracing of inputs to CLCX using a transgenic mouse line. Immunohistochemical staining against myelin basic protein (MBP), parvalbumin (PV), and calbindin (CB) revealed intricate fiber‐architectural patterns enabling precise delineations of CLCX and its subregions. Myelinated fibers were abundant dorsally in CL but absent ventrally, whereas PV expressing fibers occupied the entire CL. CB staining revealed a central gap within CL, also visible anterior to the striatum. The *Nr2f2, Npsr1*, and *Cplx3* genes expressed specifically within different subregions of the CLCX, and *Rprm* helped delineate the CL‐insular border. Furthermore, cells in CL projecting to the retrosplenial cortex were located within the myelin sparse area. By combining own experimental data with digitally available datasets of gene expression and input connectivity, we could demonstrate that the proposed delineation scheme allows anchoring of datasets from different origins to a common reference framework.

## Graphical abstract

The combinatorial expression patterns of multiple, methodologically different markers resulted in a comprehensive guide to delineate the claustrum complex (CLCX) in mice. The CLCX can be reliably divided into four subregions, all of which exhibit distinct patterns in fiber‐ and cytoarchitecture, genetic markers, and connectivity patterns. Immunohistochemical markers for myelin basic protein, parvalbumin, and calbindin‐D28 in the same tissue revealed an intricate architectural organization within the CLCX that defined its borders. Image approximation allowed to subsequently relate the fiber‐architectural framework with genetic marker data and connectivity.

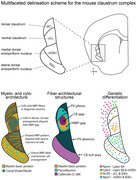

## Significance Statement

Mice are a highly tractable model for studying the claustrum complex (CLCX). However, without a consensus on how to delineate the CLCX in rodents, comparing results between studies is challenging. It is therefore important to expand our anatomical knowledge of the CLCX, to match the level of detail needed to study its functional properties. To improve and expand upon preexisting delineation schemes, we used the combinatorial expression of several markers to create a comprehensive guide to delineate the CLCX and its subregions, including an online reference atlas. This anatomical framework will allow researchers to anchor future experimental data into a common reference space. We demonstrated the power of this new structural framework by combining our own experimental data with digitally available data on gene expression and input connectivity of the CLCX.

**List of anatomical abbreviations**
AA: Anterior amygdaloid area
ACo: Anterior cortical amygdala
AH: Anterior hypothalamus
AOD: Anterior olfactory nucleus, dorsal part
AOL: Anterior olfactory nucleus, lateral part
AOM: Anterior olfactory nucleus, medial part
AOV/P: Anterior olfactory nucleus, ventral/posterior part
APir: Amygdala piriform transition zone
Aud c: Auditory cortex
BLA: Basolateral amygdala, anterior
BLP: Basolateral amygdala, posterior
BLV: Basolateral amygdala, ventral
BMA: Basomedial amygdala
BNST: Bed nucleus of the stria terminalis
CA1‐3: Cornu ammonis 1‐3
CeA: Central amygdala
cIns: Insular cortex, caudal part
cM2: Secondary motor cortex, caudal part
cPir: Piriform cortex, caudal area
CPu: Caudoputamen
CxA: Cortex amygdala transition zone
dACC: Anterior cingulate cortex, dorsal area
DG: Dentate gyrus
DPed: Dorsal peduncular area
DPir: Deep piriform area
DTT: Dorsal tenia tecta
GP: Globus pallidus
Hb: Habenula
IEn: Intermediate endopiriform nucleus
IL: Infralimbic cortex
IPAC: Interstitial nucleus of the posterior limb of the anterior commissure
LA: Lateral amygdala
LEC: Lateral entorhinal cortex
LH: Lateral hypothalamus
LO: Lateral orbital cortex
LOT: Nucleus of the lateral olfactory tract
M1: Primary motor cortex
Mam.n: Mammillary nucleus
MEC: Medial entorhinal cortex
Med A: Medial amygdala
Meta Th: Metathalamus
MH: Medial hypothalamus
MO: Medial orbital cortex
NAcc: Nucleus accumbens
NDB: Nucleus of the diagonal band
OB: Olfactory bulb
Other BS: Other brain stem areas
P.Opt: Preoptic area
PAG: Periaqueductal grey
Par c: Parietal cortex
PaS: Parasubiculum
PCo: Posterior cortical amygdala
PER: Perirhinal cortex
PH: Posterior hypothalamus
PL: Prelimbic cortex
Pn: Pontine nuclei
POR: Postrhinal cortex
PrS: Presubiculum
rIns: Insular cortex, rostral part
rM2: Secondary motor cortex, rostral part
Rn: Raphe nucleus
rPir: Piriform cortex, rostral area
RSC: Retrosplenial cortex
Rt: Reticular formation
Sub: Subiculum
SSc: Somatosensory cortex
SIB: Substantia innominata
Sept: Septal complex
STh: Subthalamic nucleus
SN: Substantia nigra
TeA: Temporal association area
Th MNG: Thalamic midline nuclear group
Th ANG: Thalamic anterior nuclear group
Th LNG: Thalamic lateral nuclear group
Th VNG: Thalamic ventral nuclear group
Th IL: Thalamic intralaminar nuclear group
Th MD: Thalamic mediodorsal nucleus
Tu: Olfactory tubercle
VLO: Ventrolateral orbital cortex
VO: Ventral orbital cortex
vACC: Anterior cingulate cortex, ventral area
VEn: Ventral endopiriform nucleus
VTT: Ventral tenia tecta
VTA: Ventral tegmental area
Vis v: Visual cortex
VP: Ventral pallidum
ZI: Zona incerta

## 1 INTRODUCTION

There is a rapidly growing interest in understanding the functional roles of the claustrum complex (CLCX), as it may be involved in key elements of cognition like attention (Atlan et al., 2018; Goll et al., 2015), multimodal integration (Crick & Koch, 2005; Shelton et al., 2022), and memory processing (Behan & Haberly, 1999; Witter et al., 1988). All these functions likely depend on interactions of the intrinsic circuitry with the extensive connectivity between the CLCX and prefrontal, sensory, and parahippocampal regions (Atlan et al., 2017; White et al., 2017; Zingg et al., 2018). To conduct fine‐scale functional investigations of brain circuitry, we need accurate anatomical definitions to integrate observations across studies. In many mammals, like primates, cats, dogs, sheep, pigs, dolphins, echidnas, and to some extent rabbits, the CLCX is encapsulated by dense myelinated fibers both medially by the external capsule, and laterally by the extreme capsule (Ashwell et al., 2004; Buchanan & Johnson, 2011; Cozzi et al., 2014; Gattass et al., 2014; Kowianski et al., 1999; Pham et al., 2019; Pirone et al., 2021, 2015, 2018; Rahman & Baizer, 2007; Reynhout & Baizer, 1999; Wojcik et al., 2002). However, in animals like mice, rats, fruit bats, and pangolins, the extreme capsule is only rudimentary, making it difficult to located claustral borders by white matter alone (Bruguier et al., 2020; Imam et al., 2022; Kowianski et al., 1999; Morello et al., 2022; Orman et al., 2017). Moreover, it has been a longstanding challenge, in particular in the most commonly used rodent models, to define clear and robust schemes for potential subdivisions of the CLCX (Watson & Puelles, 2017). In this article, we investigated the combinatorial use of expression patterns of multiple markers to develop a multifaceted anatomical description of the CLCX in mice.

The rodent CLCX is situated deep to the insular and piriform cortex and is generally considered to have two main subdivisions, the claustrum (CL) and the dorsal endopiriform nucleus (DEn) (Kowianski et al., 1999), although the functional and ontogenetic relationship between these regions has long been a matter of debate (Binks et al., 2019; Watson & Puelles, 2017). We follow the recent proposal to classify the CL and DEn as subdivisions of the same complex, based on anatomical and genetic similarities (Smith et al., 2019). One strategy for defining the borders of these regions is with cytoarchitectural staining methods like Cresyl Violet, where an increased cell density in the CLCX separates it from adjacent cortex, and the CL is distinct from the DEn due to its darker cell staining (Behan & Haberly, 1999; Druga et al., 2014; Paxinos & Franklin, 2019). Furthermore, uncertainty exists on whether the CL could be further subdivided into a dorsal and ventral domain or not (Binks et al., 2019; Mathur et al., 2009). The exact locations of all these borders are difficult to establish using cytoarchitecture alone, due to the complexity of cytoarchitectural patterns that converge in this part of the brain. Consequently, recent efforts to define these borders have benefited from included one or more additional markers, like distribution patterns of certain proteins or genes (Graf et al., 2020; Hoerder‐Suabedissen et al., 2023; Morello et al., 2022; Wang et al., 2023).

Perhaps the most established immunohistochemical (IHC) marker for the CL is parvalbumin (PV), which labels a dense plexus of neuropil that distinguishes it from surrounding structures (Davila et al., 2005; Druga et al., 1993; Mathur et al., 2009; Real et al., 2003; Wojcik et al., 2006). Conversely, IHC markers like calbindin‐D28 (CB), calretinin (CR), somatostatin (SST), myelin basic protein (MBP), and tyrosine hydroxylase show a paucity of labeling in the CL area that can also help define its borders (Barbier et al., 2017; Borroto‐Escuela & Fuxe, 2020; Celio, 1990; Davila et al., 2005; Druga et al., 2014; Graf et al., 2020; Marriott et al., 2021; Real et al., 2003; Wang et al., 2017, 2023). The chemo‐architecture of the DEn is not as well characterized but shows an elevated expression of oxytocin and dopamine receptors (Biggs & Hammock, 2022; Lothmann et al., 2021; Yoshida et al., 1988). A complete characterization of all these markers is beyond the scope of this article, and instead we have selected a few markers, whose combined expression in the same tissue would yield a robust fiber‐ and cytoarchitectural basis for delineating the CLCX.

Several genetic markers show an elevated expression in the CLCX that have been used to delineate its borders. Genes like *Lxn*, *Gng2*, *Gnb4*, *Nurr1*, *Ntng2*, and *Synpr* express generally within the CLCX, whereas *Cdh8*, *Ctgf*, *Cplx2*, *Cplx3*, and *Npsr1* show more subregional specificity, and the *Crym* gene expresses around but not inside the CL (Arimatsu et al., 2003, 1994; Binks et al., 2019; Clark et al., 2011; Davila et al., 2005; Dillingham et al., 2017; Fang et al., 2021; Hatanaka et al., 1994; Heuer et al., 2003; Hoerder‐Suabedissen et al., 2023; Mathur et al., 2009; Medina et al., 2004; Watakabe et al., 2014; Watson & Puelles, 2017). Typically these markers are analyzed in juxtaposition, although a recent article used a novel approach to co‐label several genetic markers in and around the CL within the same tissue (Erwin et al., 2021). Currently there are also a few transgenic mouse lines that show regional specificity for the CLCX, like the Gnb4‐IRES‐Cre and Ntng2‐IRES‐Cre lines labeling cells in the CL area, the Ctgf–Cre line labeling the cortical subplate as well as parts of the DEn (Wang et al., 2017), the *Egr2*‐line with labeled cells centrally in the CL (Atlan et al., 2018), the Cla–Cre line that reportedly labels a more dorsal patch of cells in the CL area (Narikiyo et al., 2020), and the GIN‐mouse line with labeled cells in the DEn (Riedemann et al., 2019).

Connectivity patterns can also be helpful to further support architectural borders, and for the rodent CL there are several examples of this. One is the dense pocket of cells projecting to the retrosplenial cortex (RSC) (Dillingham et al., 2019; Ham & Augustine, 2022; Marriott et al., 2021; Zingg et al., 2018). These RSC‐projecting cells are located centrally within the PV plexus, an organization that is often referred to as the “core and shell” model of CL (Atlan et al., 2017; Davila et al., 2005; Marriott et al., 2021; Real et al., 2003), which has also been described based on genetic markers (Erwin et al., 2021), although the existence of a CL shell has been debated (Mathur et al., 2009). Projections to and from the anterior cingulate cortex (ACC) and primary motor cortex have also been used to locate the CL, due to the dense reciprocal and bilateral projections seen between either of these regions and the CL (Alloway et al., 2009; Fillinger et al., 2017, 2018; Smith & Alloway, 2010; Smith et al., 2012; White et al., 2017). Other projections, like those arising from anterior insular cortex and parts of the amygdala, show an opposite expression pattern with dense labeling surrounding the CL (Majak et al., 2002; Qadir et al., 2018). In addition to this, dorsoventral gradients in claustral input and output connectivity have been reported (Atlan et al., 2017; Kitanishi & Matsuo, 2017; Marriott et al., 2021). To our knowledge there are no comparable “connectivity markers” for the DEn, despite it having a vastly different connectivity pattern compared to the CL (Behan & Haberly, 1999; Majak et al., 2002; Watson et al., 2017). Overall, the projection patterns to and from the CLCX have a complex organization, and thus it may be that connectivity by itself is not sufficient to define claustral borders.

In the present study, we describe a comprehensive guide for delineating the mouse CLCX based on the combined expression patterns of several different markers. We improve upon preexisting delineation schemes by analyzing multiple markers in the same tissue and use this as a basis onto which other data could be anchored. As such we could relate both our own experimental data and publicly available gene expression and input connectivity data to the same architectural framework. Together, we discovered patterns that reveal what we consider a robust, and versatile definition of the mouse CLCX and its subdivisions that will be of use to future functional studies on this region.

## 2 MATERIALS AND METHODS

### 2.1 Animal care and husbandry

Experiments were conducted at the Kavli Institute for Systems Neuroscience at the Norwegian University of Science and Technology (NTNU), Trondheim, and the Department of Physiology, Anatomy & Genetics, at the University of Oxford. Animals were group housed in environmentally enriched cages and given ad libitum access to food and drink. Animals housed at the University of Oxford were kept at a normal 12:12 h day/night cycle, while at NTNU the day/night cycle was reversed. Both male and female animals were used, and we did not notice any variation in our data pertaining to the sex of the animal. All procedures involving animals were done in accordance with guidelines of the Federation of European Laboratory Animal Science Association, and local authorities at NTNU and the University of Oxford. Surgical procedures performed at the University of Oxford were carried out under license from the UK Home Office in accordance with the Animal (Scientific Procedures) Act 1986. Experiments carried out at NTNU were approved by the Norwegian Food Safety Authority.

### 2.2 Transgenic mouse lines

Two transgenic mouse lines were used in this study: The CLCX‐enhancer‐driven gene expression (CLCX‐EDGE) line and Tre‐Tight‐THAG line. The cross between these two, *CLCX‐EDGE:TRE‐Tight‐THAG*, was used in monosynaptic rabies tracing experiments. The CLCX‐EDGE transgenic mouse line, originally called medial entorhinal cortex (MEC)‐13‐53D (Blankvoort et al., 2018), expresses tetracycline transactivator (tTA) protein within a subpopulation of cells that are largely confined to the CLCX. The Tre‐Tight‐THAG transgenic line was generated using the following steps. The gene sequences of avian‐specific tumor virus receptor A (TVA), hemagglutinin tag (HA), and challenge virus standard‐11 glycoprotein (CVS11G) separated by 2A elements were inserted between Xma1 and Mlu1 restriction sites in the pTT2 construct previously described in Weible et al. (2010). After the sequence verification, the resulting construct pTT2‐TVA‐2A‐2xHA‐2A‐CVS11G was linearized, run on 1% agarose gel, and purified using Zymoclean Gel DNA Recovery Kit (Zymo research, D4001) as per protocol. The transgenic mouse facility of the University of Oregon carried out pronuclear injections to create the TRE‐Tight‐THAG line. In CLCX‐EDGE:TRE‐Tight‐THAG transgenic animals, rabies glycoprotein and TVA receptors were conditionally expressed in tTA producing cells.

### 2.3 General surgical procedures and tissue acquisition

General anesthesia was induced with 5% isoflurane (IsoFlo vet) prior to surgical procedures. A continuous flow of isoflurane and oxygen was administered throughout surgeries and adjusted to maintain stable anesthesia. Animals were placed in a stereotaxic frame while resting on a heating pad at 37°C for the duration of the procedure. Analgesics were given prior to surgery, either through subcutaneous injections of metacam (1 mg/kg) and temgesic (0.1 mg/kg) or intraperitoneal injections of metacam (5 mg/kg) and buprenorphine (0.1 mg/kg). Saline injections were administered during the surgery to avoid dehydration. The incision area was disinfected with iodine and subcutaneously injected with a local anesthetic (Marcaine 1 mg/kg or bupivacaine) for 2 min before the initial incision. The cranium was manually leveled between bregma and lambda, and between the left and right hemisphere. Craniotomies were performed using a dental drill and were exclusively done in the right hemisphere unless otherwise noted. Pulled glass pipettes (World Precision Instruments, Glass Replacement 1.14 mm 3.5″, item no. 504949) were used for all injections. Dorsoventral depth was measured from the surface of the pia. Pipettes remained in place for 10 min before retraction. Postoperative analgesia was administered 7–12 h after surgery, and animals were given easily ingestible food (oat porridge). Further analgesia was administered when necessary. Prior to tissue collection, animals were deeply anaesthetized with isoflurane and given a lethal intraperitoneal injection of pentobarbital (0.1 mL ip of a solution of 100 mg/mL). Animals were carefully observed until breathing ceased, and motor and eye blinking reflexes were gone, at which point transcardial perfusion was performed using a peristaltic pump (MasterFlex) to pump Ringer saline solution followed by 4% paraformaldehyde (Sigma Aldrich) in 0.125 M phosphate buffer through the circulatory system. Brains were postfixed in 4% paraformaldehyde overnight and then transferred to a cryoprotective solution containing 20% glycerol and 2% dimethyl sulfoxide in 0.125 M phosphate buffer.

### 2.4 Antibody characterization

Several primary antibodies were used in the present article, all of which were characterized and tested by their respective manufacturers. We tested several markers to label myelinated axons and settled on using an antibody against MBP. This antibody has been shown to label non‐phosphorylated parts of the neurofilaments in human brain preparations (Evers & Uylings, 1997), and labeled four isoforms of MBP at 1:500 concentration in western blot analysis of mouse tissue lysate, as documented by the manufacturer. We used two different antibodies for both PV and CB depending on host‐organism availability in each procedure. Both PV antibodies recognized the 12 kDa PV protein in multiple species, including mice, as described by the manufacturers: The Sigma antibody was characterized using the Sigma ImmunoTypeTM Kit as well as a double diffusion immunoassay, and the antibody from Swant was characterized using sodium dodecyl sulfate gel electrophoresis. CB antibodies were shown by the manufacturer to recognize the 28 kDa calbindin‐D28k protein in multiple species including mice; the antibodies were tested by the manufacturer using two‐dimensional gel electrophoresis. Some experiments included a NeuN antibody, which has been extensively characterized in the literature and binds to the neuron‐specific protein NeuN found in most neuronal cell types. Rabies tracing data were stained using antibodies against the 2A linker protein, tested by the manufacturer with western blot analysis, and a red fluorescent protein (RFP) antibody, tested by the manufacturer with IHC on cultured human cells. Detailed information of all antibodies used can be found in Table 1.

**TABLE 1:** Antibody information.

Antibody	Manufacturer	Catalog no.	RRID	Dilution
**Primary antibodies**				
Guinea pig anti‐NeuN	Millipore	ABN90P	AB_2341095	1:1000
Mouse anti‐myelin basic protein	Millipore	NE1019	AB_2140491	1:1000
Mouse anti‐parvalbumin	Sigma‐Aldrich	P3088	AB_477329	1:1000
Mouse anti‐calbindin‐D28k	Swant	300	AB_10000347	1:3000
Rabbit anti‐parvalbumin	Swant	PV27	AB_2631173	1:1000
Rabbit anti‐calbindin‐D28k	Swant	CB38	AB_10000340	1:3000
Rabbit anti‐2A peptide	Millipore	ABS31	AB_11214282	1:2000
Rat anti‐red fluorescent proteins	ChromoTek	5f8‐100	AB_2336064	1:500
**Secondary antibodies**				
Goat anti‐guinea pig Alexa Fluor 647	Thermo Fischer Sci.	A21450	AB_141882	1:400
Goat anti‐mouse Alexa Fluor 488	Thermo Fischer Sci.	A11001	AB_2534069	1:400
Goat anti‐mouse Alexa Fluor 546	Thermo Fischer Sci.	A11003	AB_141370	1:400
Goat anti‐rabbit Alexa Fluor 488	Thermo Fischer Sci.	A11008	AB_143165	1:400
Goat anti‐rabbit Alexa Fluor 546	Thermo Fischer Sci.	A11010	AB_2534077	1:400
Goat anti‐rabbit Alexa Fluor 635	Thermo Fischer Sci.	A31576	AB_1500684	1:400
Goat anti‐rat Alexa Fluor 546	Thermo Fischer Sci.	A11081	AB_2534125	1:400

### 2.5 Histology and immunohistochemistry

Brains were sliced into 40 μm thick coronal sections using a freezing sliding‐microtome (Thermo Scientific HM 430), kept at approximately −40°C. Four equally spaced series of sections were collected for each brain, allowing for multiple histological staining procedures on sections of the same brain. Various IHC procedures were carried out with standard protocols for free floating brain sections. Permeabilization of brain sections was done using a phosphate buffer solution containing 0.5% Triton X‐100 (Merck KGaA). Blocking was done with 5% normal goat serum (Abcam, #ab7481) at room temperature. Primary antibody incubation was done for at least 48 h at 4–5°C. Secondary antibody incubation was done for 1–2 h at room temperature. Sections were mounted onto microscope slides (Menzel‐Gläser SuperFrost Plus) and left to dry overnight. On the following day, sections were cleared for 10 min in Toluene (BDH Prolabo) and coverslipped in a mixture of Toluene and Entellan (Merck KGaA). Another mounted series was used for Nissl staining with Cresyl Violet (Sigma Life Science, C5042), after tissue clearing in Xylene (Merck KGaA) and multiple steps of rehydration from 100% to 50% ethanol. Some experiments involved IHC staining followed by de‐coverslipping and Nissl staining with Cresyl Violet. Two animals stained against MBP and CB were originally used to characterize the crossbreed of transgenic lines CLCX‐EDGE and tetO‐Chrimson, but as they showed no apparent variation in expression of these markers they were included in this dataset. We included two animals from older experiments where only the staining of PV and MBP was used, which is why the number of CB‐stained animals is less than PV and MBP.

### 2.6 Neuroanatomical tract tracing

Two different injection strategies were used in this study (Table 2). CLCX‐EDGE::TRE‐Tight‐THAG transgenic mice (aged 11–23 weeks) were unilaterally injected with EnvA‐pseudotyped, G‐protein deleted CVS‐N2c recombinant rabies virus expressing tdTomato (RABV‐tdTomato, 10^7^ functional virus particles/mL) at one or two coordinates in the CLCX. The RABV‐tdTomato virus was provided by Dr. Rajeevkumar R. Nair and produced at the Viral Vector Core at the Kavli Institute for Systems Neuroscience. RABV‐tdTomato was injected at 10–20 μL/s. The brains from these animals were collected 10–14 days after the injection following the procedure described before. C57BL6J mice (aged 3–5 weeks) were unilaterally injected with cholera toxin subunit B (CTB (Recombinant) Alexa Fluor 647 Conjugate [0.1% wt/vol, Thermo Fisher C34775]) in the RSC. CTB was injected at 5–10 nL/s. CTB injected animals were allowed to recover for a minimum of 5 days before brains were collected.

**TABLE 2:** Injection coordinates.

			Injection coordinates (mm)		
Animal ID	Injection volume (nL)	Sex	Anteroposterior	Mediolateral	Dorsoventral
**Rabies tracing**					
Rb1	750/750	F	+1.33/+0.11	+3.2/+4.0	−2.8/−2.8
Rb2	500/600	F	+1.33/+0.11	+3.4/+4.1	‐2.9/−2.8
Rb3	500/500	F	+1.33/+0.11	+3.2/+4.0	−2.9/−2.8
Rb4	500/500	M	+1.33/+0.11	+3.2/+4.0	−2.9/−2.8
Rb5	150/150	F	+1.33/+0.11	+3.2/+4.1	−2.9/−3.1
Rb6	800	M	+1.33	+3.2	−2.9
Rb7	600/600	F	+1.33/+0.11	+3.2/+4.0	−2.8/−2.8
Rb8	500/500	M	+1.33/+0.11	+3.2/+4.3	−2.9/−2.8
Rb9	300/300	F	+1.33/+0.11	+3.1/+3.7	−3.2/−3.2
Rb10	400/200	M	+1.33/+0.11	+3.1/+3.8	−3.2/−3.2
Rb11	800	F	+1.33	+3.1/+3.7	−3.2/−3.2
Rb12	200	M	+1.33	+3.3	−2.6/−2.6
Rb13	100	F	−0.7	−0.7^a^	−2.6/−2.6
**CTB tracing**					
CTB1	80	M	−3.0	+0.5	−1.0
CTB2	80	M	−3.0	+0.5	−1.0
CTB3	80	M	−3.0	+0.5	−1.0
CTB4	80	M	−3.0	+0.5	−1.0
CTB5	80	M	−3.0	+0.5	−1.0
CTB6	80	M	−3.0	+0.5	−1.0

^a^ Measured from the lateral edge of the cranium.

### 2.7 Quantification of brain‐wide monosynaptic inputs to the claustrum complex

Rabies injected brains were IHC stained using antibodies against the 2A linker protein found in tTA expressing cells, and an RFP tag expressed by the rabies virus. Cell counting was done with *Neurolucida 2014* software (MBF Bioscience). Following cell quantification, each slide was de‐coverslipped and stained with Cresyl Violet. Images of each section were then superimposed onto the contours of the sections and the position markers of counted cells, allowing close approximation of the fluorescent and cytoarchitectural images resulting in the accurate position of input cells throughout the brain. Microsoft Excel and custom‐made scripts in MATLAB were used to analyze and visualize the quantification of the tracing data.

### 2.8 Image acquisition and processing

Fluorescence and brightfield images were acquired using a slide scanner with a Plan‐Apochromat 20X/0.8 NA M27 objective, resulting in a resolution of 0.325 μm/pixel (Axio Scan.Z1, ZEISS). A series of higher resolution fluorescent images were obtained with a confocal microscope (LSM 880, ZEISS). Images were post‐processed with ZEN Black 2.1 SP2, ZEN Blue 2.3 Lite, and Adobe Photoshop to enhance the signal quality. All image processing was applied to the entire image. CTB images were acquired using an Olympus FV3000 confocal laser scanning microscope and post‐processed in ImageJ and Python 3.7.

### 2.9 Rostrocaudal landmarks

We selected five landmarks to locate different rostrocaudal levels of the CLCX. The landmarks were approximately at the same dorsoventral level as the CLCX, to mitigate variation from slight differences in the sectioning plane. Distance to bregma was estimated by comparison to a reference atlas (Paxinos & Franklin, 2019). The far rostral landmark was selected where the orbital cortex merges with the olfactory cortex (B+2.09). As the rostral landmark, we selected the caudal‐most section displaying the dorsal peduncular area, positioned between the corpus callosum and the septal complex (B+0.97). Roughly at the midpoint of the CLCX, the central landmark was chosen as the point where the anterior commissure joins at the midline (B+0.13). The caudal landmark was selected at the rostral‐most section showing the basolateral amygdala (B−0.59). Finally, the far caudal landmark was chosen where the optic tract joins with the internal capsule (B−1.43). Notably, the far rostral and far caudal landmarks do not represent the rostral and caudal edges of the CLCX.

### 2.10 Delineation references

We used a combination of research articles and atlases for our cytoarchitectural delineations of brain regions. Table 3 lists articles that were used, and for which borders they were used. Other regions were delineated based on the Paxinos & Franklin Mouse reference atlas (Paxinos & Franklin, 2019).

**TABLE 3:** Delineation references.

Border(s) described	References
Prefrontal cortex	van de Werd et al. (2010)
Hippocampal formation	Witter (2012), Witter and Amaral (2004)
Parahippocampal formation	Beaudin et al. (2013), Burwell (2001), Insausti et al. (1997)
Other cortical borders	Hovde et al. (2019), Malmierca and Ryugo (2012), Watson (2012a, 2012b), Young et al. (2012)
Subcortical areas	Root et al. (2015)

### 2.11 Data‐approximation by image‐warping

Images were collected from online in situ hybridization (ISH) and tracer databases (2006 Allen Institute for Brain Science, ISH Data, Available from: https://mouse.brain‐map.org/, 2017 Allen Institute for Brain Science, Projection Dataset, Available from: https://connectivity.brain‐map.org/), and used in an image approximation procedure. Hyperlinks to the experiments we used can be found in Table 4. For each experiment, we downloaded images of sections with rostrocaudal position closest to our selected landmarks (see before). All images were converted to gray scale. The BigWarp function in ImageJ was used to align each brain section to a reference by creating a set of transformation coordinates (Bogovic et al., 2016; Schneider et al., 2012). An alignment frame was drawn onto each section using vector graphics in Adobe Illustrator to assign the necessary number of anchor points to allow for accurate transformation. A few cortical borders were indicated, and equidistant points between these borders were then marked to divide the cortex into multiple “bins” (*n* = 4 bins for insular cortex, *n* = 4 bins for piriform cortex, and *n* = 8 bins from somatosensory cortex [SSc] to cingulate cortex). ISH data were thresholded using the inbuilt function in ImageJ to remove background and allow the data to be superimposed onto the reference image. Brightness and contrast were adjusted slightly to maintain similar conditions for the thresholding. Each thresholded section was compared to the original ISH image to confirm correct representation of the expression.

**TABLE 4:** Online dataset references.

Database	Hyperlink
Allen in situ hybridization database ‐ Genetic markers	
Ccdc3	https://mouse.brain‐map.org/experiment/show/75651160
Synpr	https://mouse.brain‐map.org/experiment/show/1862
Nr2f2	https://mouse.brain‐map.org/experiment/show/112646890
Rxfp1	https://mouse.brain‐map.org/experiment/show/70562124
Matn2	https://mouse.brain‐map.org/experiment/show/73817421
Npsr1	https://mouse.brain‐map.org/experiment/show/70560288
Gsta4	https://mouse.brain‐map.org/experiment/show/77278951
Col23a1	https://mouse.brain‐map.org/experiment/show/73636092
Meis2	https://mouse.brain‐map.org/experiment/show/1231
Tle4	https://mouse.brain‐map.org/experiment/show/73521809
Tpbg	https://mouse.brain‐map.org/experiment/show/76085743
Cdh24	https://mouse.brain‐map.org/experiment/show/70231307
Gm1441	https://mouse.brain‐map.org/experiment/show/72109262
Ctgf	https://mouse.brain‐map.org/experiment/show/79556634
Cplx3	https://mouse.brain‐map.org/experiment/show/70928340
Galnt10	https://mouse.brain‐map.org/experiment/show/70814342
Itga5	https://mouse.brain‐map.org/experiment/show/74882636
Brinp3	https://mouse.brain‐map.org/experiment/show/70927812
Ighm	https://mouse.brain‐map.org/experiment/show/72109389
Fezf2	https://mouse.brain‐map.org/experiment/show/75651163
Rprm	https://mouse.brain‐map.org/experiment/show/75651169
Col12a1	https://mouse.brain‐map.org/experiment/show/73817431
Neurod6	https://mouse.brain‐map.org/experiment/show/698
Tmem163	https://mouse.brain‐map.org/experiment/show/71670711
Chst11	https://mouse.brain‐map.org/experiment/show/73931625
Sstr2	https://mouse.brain‐map.org/experiment/show/77371821

Allen projection database ‐ Injection sites	
Rn, Pn	https://connectivity.brain‐map.org/projection/experiment/310176384
Rn, Pn, PAG	https://connectivity.brain‐map.org/projection/experiment/301732962
Rn, PAG	https://connectivity.brain‐map.org/projection/experiment/301765327
Rn, PAG	https://connectivity.brain‐map.org/projection/experiment/183562831
Rn, PAG	https://connectivity.brain‐map.org/projection/experiment/272699357
MEC	https://connectivity.brain‐map.org/projection/experiment/518745840
MEC	https://connectivity.brain‐map.org/projection/experiment/557199437
MEC, PrS	https://connectivity.brain‐map.org/projection/experiment/286484879

### 2.12 Fluorescence profile analysis

**FIGURE 1:**
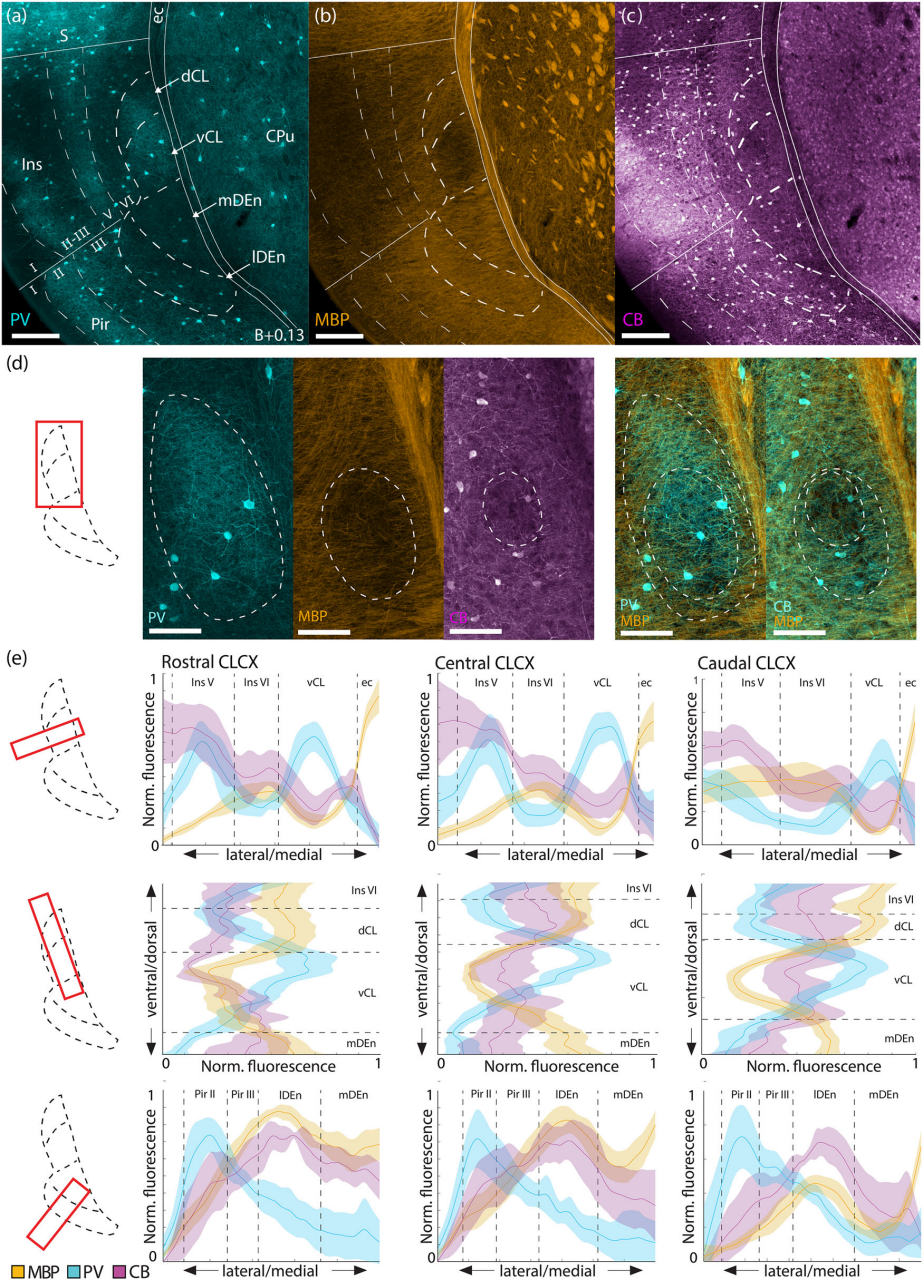
Fiber‐architectural characterization of the claustrum complex. (a–c) Reappraisal of borders to claustrum complex (CLCX) based on overlapping patterns in tissue triple‐stained for parvalbumin (PV), myelin basic protein (MBP), and calbindin (CB) in female C57 mouse. Scale bars measure 200 μm. (d) Delineations of the PV‐plexus, MBP‐gap, and CB‐gap in the same tissue. The two rightmost panels show merged expression of MBP/PV and MBP/CB expression. Both PV and CB labelings are pseudo‐colored cyan to increase visibility. Scale bars measure 100 μm. (e) Average fluorescence traces of PV (*n* = 5 mice) MBP (*n* = 5 mice), and CB (*n* = 3 mice) along mediolateral and dorsoventral gradients, at rostral (B+0.97), central (B+0.13), and caudal (B−0.59) levels of the CLCX. Bilateral measurements were made in each brain. Dashed lines indicate approximate borders based on the combined profile patterns. Fluorescence measures were normalized across images using min–max scaling. Shaded areas indicate 95% confidence intervals. Images at each rostrocaudal level were warped to a reference section. Abbreviated terms are explained in the list of abbreviations. Confocal images in (a–d) show maximum intensity projections of z‐stacks taken at 10× magnification.

Average fluorescence traces were measured in ImageJ using the “Plot profile” function to produce average signal intensity traces within a rectangular region. In fiber‐architectural comparisons (Figure 1e), bilateral claustra were analyzed in each animal (*n* = 5 animals stained against PV and MBP and *n* = 3 animals stained against CB). For these measurements, a rectangular area was placed in approximately the same location in each section, as determined by the image warping pipeline described before. For Figure 5, images across mice (*n* = 6) and within matched representative sections were aligned to the max CTB signal, cropped to the same area and rotated along the external capsule. Images were then normalized and averaged along the horizontal and vertical axes to produce fluorescence traces. The fluorescence profiles were processed in MATLAB or Python and smoothed using a Gaussian filter. The intensity of the signal was scaled using min–max normalization.

### 2.13 Data availability statement

The data that support the findings of this study are openly available in an Open Science Framework repository, at https://osf.io/sdwrv/. This repository contains a reference atlas with delineations of the entire CLCX in closely spaced coronal sections (80 μm). Each panel in the atlas shows fluorescent staining of PV and MBP, in addition to Cresyl Violet staining of the same section. The Cresyl Violet images were aligned to the fluorescent images manually in Adobe Illustrator. Further histological data and delineations are available upon request.

### 2.14 Statistical analyses

Custom made scripts in MATLAB and Python were used for data analysis and quantification. Fluorescence profiles were analyzed as previously described, using basic programming functions to display mean values with a 95% confidence interval. In the rabies tracing dataset, percentage values were measured per animal; input cells within the CLCX were not included in these calculations. Mean percentage values were measured across animals, for each individual region, and represented including the standard error of the mean.

## 3 RESULTS

### 3.1 Fiber‐ and cytoarchitecture of the claustrum complex

The combined IHC data from PV, MBP, and CB labeling revealed fiber‐architectural patterns in the CLCX suggesting the presence of four distinct domains (Figure 1a–c). Patterns in MBP labeling indicated that the CL can be divided into a dorsal and ventral subregion (dCL and vCL, respectively). The PV and CB patterns pointed more to a center‐surround organization of the CL, which also helped to define its perimeter. The fiber‐architectural patterns for all three markers led us to divide DEn into a medial and lateral subunit (mDEn and lDEn, respectively).

MBP staining revealed an area with reduced density of myelinated fibers ventrally in the CL, distinct both from a dorsal patch of dense labeling in CL and from a dense plexus in layers 5 and 6 of insular cortex (Figure 1b). Dorsal to this “MBP‐gap,” myelinated fibers extended from the external capsule, moving diagonally toward the insular cortex. The transition from these diagonal fibers to the MBP‐gap was used as the main indicator of the dCL–vCL border. Surprisingly, the MBP‐gap did not fully align with the dense PV‐plexus but only matched with a ventral part of it. However, dorsal parts of the PV‐plexus aligned with the patch of MBP‐labeled fibers extending diagonally from the external capsule. The dense PV‐plexus in the CL contrasted with an absence of labeling in L6 of the insular cortex and in the mDEn (Figure 1a). In general, this plexus provided a good strategy for defining the borders of the CL with the overlying cortex and the DEn. Note that in each brain, we observed variations in how far the PV‐plexus extended dorsoventrally within the CL, and some sections had a PV‐plexus that mainly occupied a central part of the CL. The combination of PV and MBP labeling was eventually used to define the border between the CL and insular cortex.

CB‐labeled processes formed a ringlike plexus in the CL, with a sparsely labeled central gap (Figure 1c), though in each brain we saw variation in how clear this gap was. Additionally, CB‐staining labeled a laminar plexus in layer 6 of insular cortex, which appeared distinct from the CL at central and caudal levels, but less so at rostral levels. The CL‐insular border was also visible in CB‐labeling, albeit not as clearly as in the other markers. Note that although the PV‐plexus, MBP‐gap, and CB‐gap appeared similar in shape, they were spatially misaligned within the CL (Figure 1d). The PV plexus extended dorsally past the MBP‐gap, occupying both the dCL and the vCL, while the CB‐gap aligned with a central region of the PV‐plexus and was located dorsally in the vCL. This is relevant when using only one of these markers to describe experimental data on the CL.

In the DEn, the division into a lateral and medial domain was indicated by all three markers. This was most easily seen in the case of CB staining where the lDEn showed dense immunoreactivity, both resulting from somatic and neurite labeling; CB‐labeling in mDEn was considerably sparser. With regards to MBP staining, the mDEn displayed a characteristic striped pattern, separating it from the homogenous labeling seen in the lDEn. The PV labeling was the least discriminative with only the lDEn displaying sparse PV‐labeling, which was also not clearly visible in all the material we assessed.

The consistency of our fiber‐architectural borders was subsequently assessed along the rostrocaudal axis and across animals by measuring average fluorescence intensity profiles of PV, MBP, and CB immunoreactivity (Figure 1e). Brain sections at the rostral, central, and caudal landmarks were selected from each animal, and aligned to a reference section using the image warping pipeline described in the methods section. Profiles from each animal represent average intensity levels of fluorescence as measured in a rectangular selection drawn across the region of interest. The combined profiles for all markers corroborate the differentiation between lDEn and mDEn, mentioned before. They further revealed an overall colocalization of the PV‐plexus, MBP gap, and the smaller CB‐gap in the CL throughout the rostrocaudal extent of the CL, with the exception of the rostral level, where a slight misalignment of the MBP‐gap and PV‐plexus was apparent, as described before.

**FIGURE 2:**
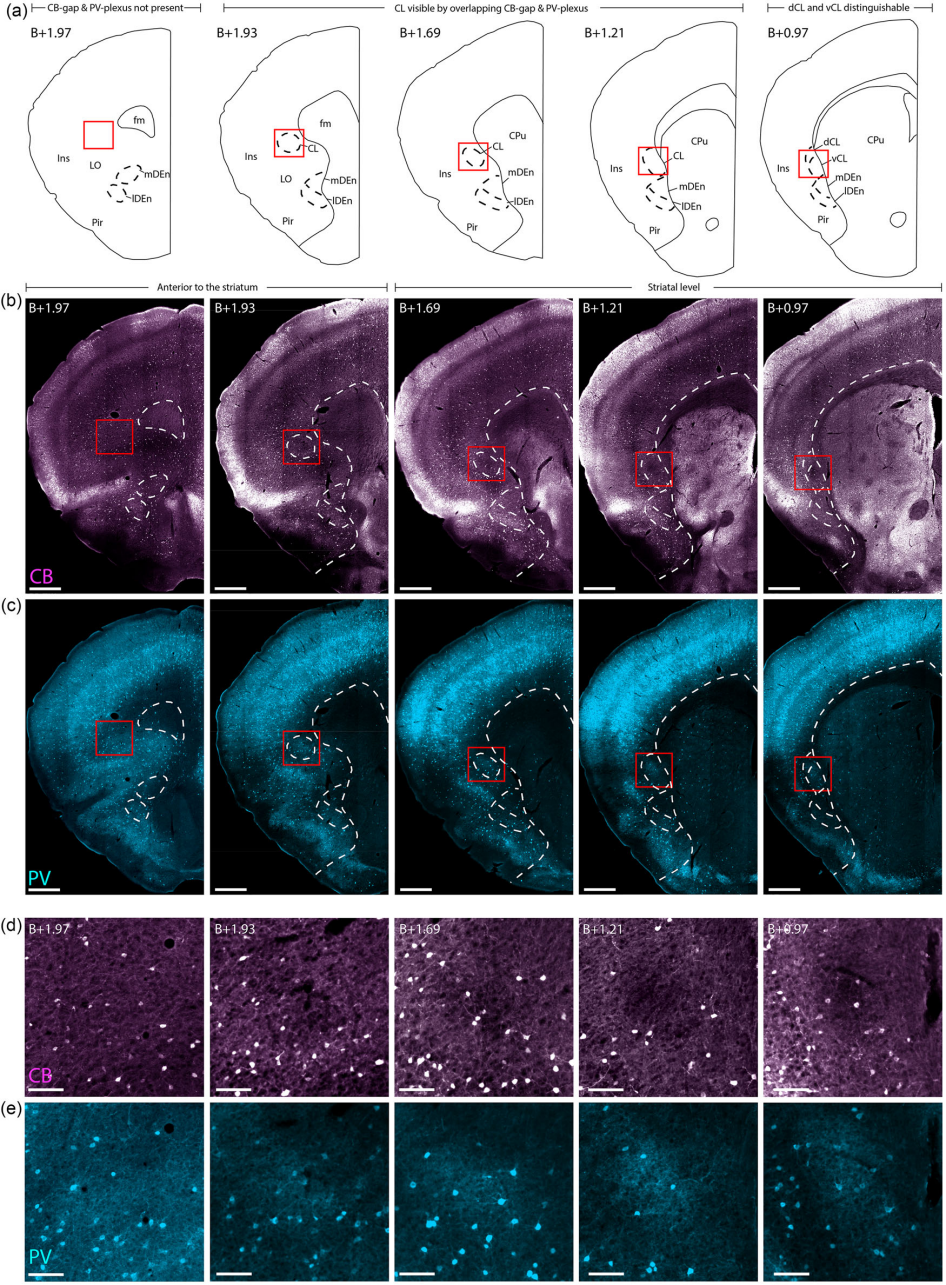
Delineating the rostral‐most parts of the claustrum in female C57 mouse. Immunohistochemical labeling of calbindin (CB) and parvalbumin (PV) in five sections, taken at gradually more rostral levels (right to left, indicated by level rostral to Bregma (B)). (a) Schematic of the histological images shown in (b–e), indicating where the striatum starts and how far rostral the claustrum (CL) can be identified. (b) Location of the CB‐gap, revealing the CL until B+1.93. (c) PV labeling of the same sections as in (b), showing the position of the PV plexus in the CL until B+1.93. (d) Insets from red squares indicated in (b), showing high magnification of the CB‐gap. (e) Insets showing the same areas as in (d) but stained for PV. Note the presence of dense PV neuropil aligned with the CB‐gap. Scale bars measure 500 μm in overview images and 100 μm in insets. All images were obtained with a slide scanner (see Section 2). Abbreviated terms are explained in the list of abbreviations.

**FIGURE 3:**
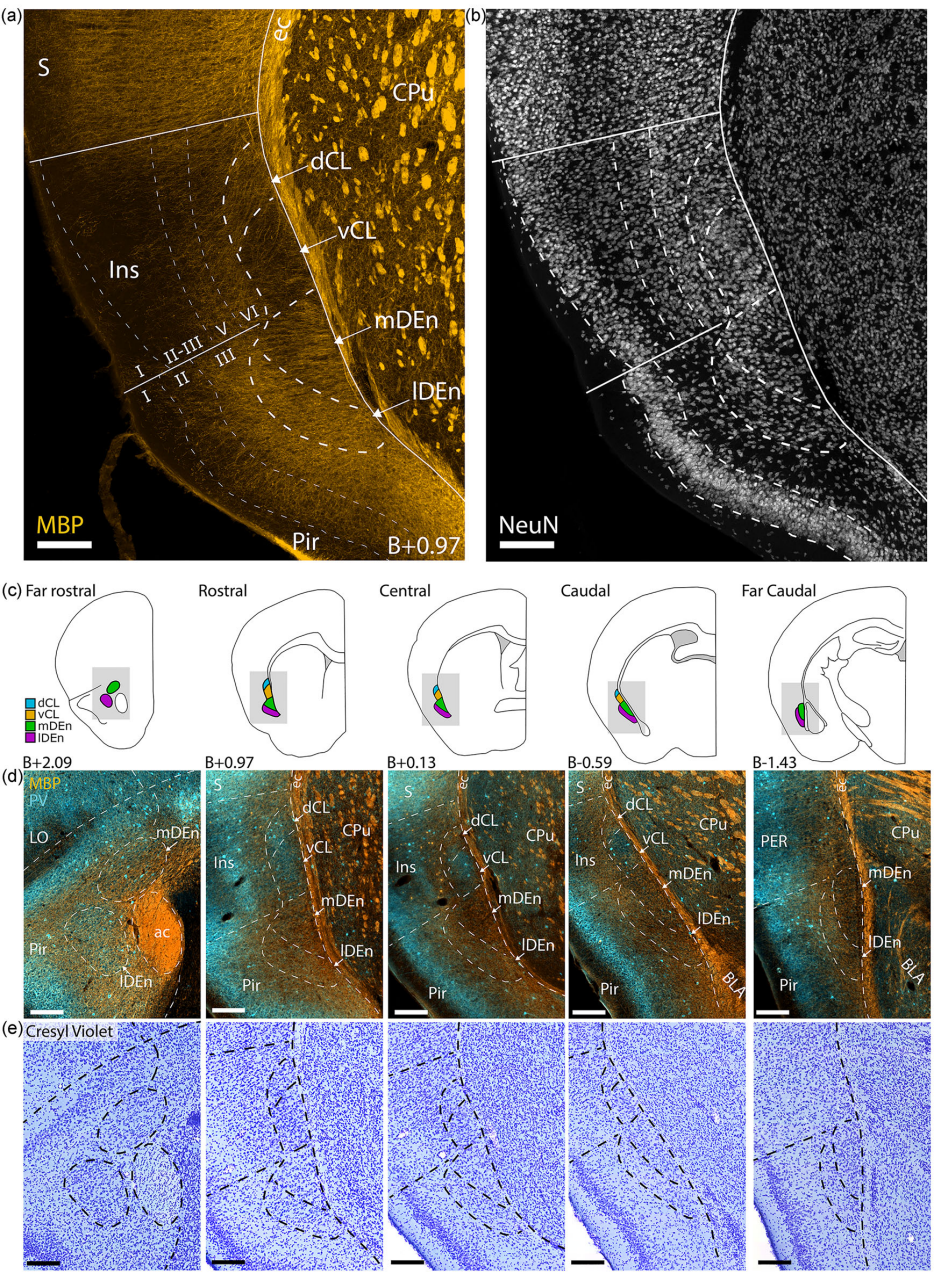
Comparison of fiber‐ and cytoarchitecture of the claustrum complex (CLCX). (a and b) Immunohistochemical staining against myelin basic protein (MBP) and NeuN in male C57 mouse. Delineations based on fiber‐architectural patterns overlap with those seen in cytoarchitecture. (c) Schematic representation of the CLCX at five rostrocaudal landmarks. Shaded areas indicate inset location in (d and e). Approximate location to bregma is shown at each landmark. (d) Co‐expression of MBP and parvalbumin (PV) at each landmark. (e) Cresyl Violet staining of the same sections shown in (d). Scale bars measure 200 μm. Abbreviated terms are explained in the list of abbreviations. Panels a and b show maximum intensity projections of z‐stacks taken with a confocal microscope at ×10 magnification, whereas panels d and e show images taken with a slide scanner of a female C57 mouse.

We next aimed to address the unresolved debate on how far CL extends rostrally, by analyzing a series of closely spaced sections (Figure 2). We found that the CB‐gap provided the clearest landmark to localize the rostral‐most parts of the CL (Figure 2b,d). By following the position of the CB‐gap gradually from the rostral landmark (B+0.97), we found that it was clearly visible until about +1.93 mm relative to bregma, but not at B+1.97. The CB‐gap aligned consistently with the PV‐plexus (Figure 2c,e). However, the PV‐plexus in far rostral sections became less distinct due to the prevalence of PV‐labeling in surrounding areas. MBP labeling showed little or no indication of the rostral‐most parts of the CL (Figure S1). Even though the combination of CB and PV turned out to be good markers to localize the rostral‐most parts of the CL, this combination did not provide a clear border between dorsal and ventral parts of the CL. Therefore, we opted not to subdivide the CL rostral to B+0.97. At levels rostral to B+1.93, the CL can no longer be identified using the current set of criteria, though DEn is still present and can be subdivided into its lateral and medial divisions (Figure 2 all left‐hand panels), and this is true even at more rostral levels at B+2.09 (Figure 3b and c–e, left‐hand panels).

To compare fiber‐ and cytoarchitecture in the CLCX, we did IHC staining against PV and MBP in addition to either NeuN or Cresyl Violet (Figure 3). Together, these experiments revealed distinct cytoarchitectural features within the fiber‐based subregions of the CLCX (Figure 3a,b). We observed that the cell‐arrangement in the CLCX was often aligned with the direction of MBP‐labeled fibers. Diagonal columns of cells could be seen in dCL, following the fibers that extended from the external capsule toward insular cortex. Note that this was not always clearly visible, but a good example can be seen in the Cresyl Violet staining in Figure 3e. Cells in vCL were arranged in a circular structure, matching the shape of the MBP gap. The mDEn showed a columnar arrangement of cells that were aligned to the stripes in MBP labeling. In comparison, the lDEn had a more laminar arrangement of cells, following the piriform cortex. These features were generally clearer in Cresyl Violet staining than NeuN labeling.

In a final series of experiments, we did immunostaining against PV and MBP followed by Nissl staining in the same tissue, to corroborate our definitions of the most rostral areas of CLCX and also to precisely define its caudal‐most parts. We also aimed to increase our resolution along the rostrocaudal axis, so we stained and mounted every second coronal section, allowing us to study the gradual change of claustral borders with close rostrocaudal increments (80 μm between sections; Figure 3c–e). With this approach, we delineated the entire rostrocaudal extent of the CLCX in a reference atlas for the mouse CLCX (https://osf.io/sdwrv/).

At far rostral levels (B+2.09), the mDEn appeared in the medial most parts of the piriform cortex, dorsal to the anterior commissure; the lDEn was distinct from the mDEn at this level and was positioned deep to piriform cortex on the lateral side of the anterior commissure (Figure 3d,e, left‐most panels). MBP‐labeled fibers were sparser in far rostral lDEn than in surrounding areas. In particular, the area between lDEn and mDEn was densely stained with MBP. Far rostral mDEn was also indicated by MBP labeling, but this was not always easy to see. In some brains, the far rostral lDEn had a clear PV plexus. Cytoarchitecturally, as seen with Cresyl Violet staining, both areas were more densely populated than the surrounding parts of piriform cortex. The CL was not visible at far rostral levels, but as shown in Figure 2, it followed the dorsoventral position of the insular cortex and was distinct from DEn in sections at levels where it was possible to differentiate the lateral orbital cortex.

At far caudal levels (B‐1.43), the lDEn covered the entire lateral border of the mDEn (Figure 3d,e, right‐most panel). The two regions were distinguished by sparse labeling of MBP in the mDEn compared to lDEn. The mDEn showed no PV labeling, whereas the lDEn showed some labeling. Cytoarchitecturally, the far caudal lDEn had a laminar appearance, while the mDEn appeared more irregular. Both regions extended as far caudal as the piriform cortex, which was gradually replaced by the lateral entorhinal cortex (LEC). The transition from lDEn and mDEn to L6 of LEC was easier to identify in MBP and PV staining than using cytoarchitectural patterns.

### 3.2 Gene expression patterns corroborate architectonic delineations

To expand upon the current toolbox of genetic markers for the CLCX, we used a list of genes acquired from a chromatin immunoprecipitation sequencing analysis of micro‐dissected CL tissue (unpublished own material). We then screened the Allen ISH database for these genes, in addition to using the in‐built differential gene search in the Allen ISH database for candidate markers in the CL, endopiriform nucleus, insular, and piriform cortices (2006 Allen Institute for Brain Science, ISH Data, Available from https://mouse.brain‐map.org/). Finally, we searched the literature for genetic markers used to identify deep cortical layers. With this approach, we looked for genes that could help delineate borders between CLCX subregions or to adjacent cortex, and thus did not include those that express throughout the CLCX. Using these criteria, we made a short list of eligible candidates (Table 5) after screening more than 1000 ISH experiments in the Allen Institute database. Note that we did not filter out genes based on their novelty to the field, resulting in some overlap between our list and prior characterizations (Dillingham et al., 2017; Erwin et al., 2021; Watson & Puelles, 2017).

**TABLE 5:** Genetic marker candidates for the claustrum complex.

Marker	Expression						
	dCL	vCL	mDEn	lDEn	Insular	Piriform	IEn/VEn
Ccdc3	+	+	–	–	++	+	–
Nr2f2	+	++	–	++	+	+	–
Rxfp1	–	+++	–	+	+	–	–
Matn2	–	++	++	–	+	+	–
Npsr1	–	+	+++	+++	–	–	–
Gsta4	–	–	+++	+	+	+	+
Col23a1	+	–	+++	++	+	+	–
Meis2	–	–	++	+	+++	++	–
Tle4	+	–	++	+	+++	–	+
Tpbg	–	–	++	+++	+++	+	++
Cdh24	–	–	++	+	++	+	–
Gm1441	+	–	++	+	+++	+	+
Ctgf	+	–	+++	++	+++	–	–
Cplx3	+	–	+++	+	+++	+	+
Galnt10	–	–	+	–	+	–	+
Itga5	–	–	+	–	+	–	–
Brinp3	–	–	–	+	+++	+	+
Ighm	–	–	–	–	+++	–	+
Fezf2	–	–	+	–	++	–	–
Rprm	+	–	–	–	+++	+++	–
Col12a1	+	–	+	–	++	–	–
Neurod6	++	+++	+++	+	+++	–	–
Tmem163	++	+++	+	+++	++	+	+
Chst11	++	+++	++	++	++	–	–
Sstr2	+	++	+	++	+	–	+

*Note*: +++: dense labeling; ++: some labeling; +: sparse labeling;—: no labeling.

**FIGURE 4:**
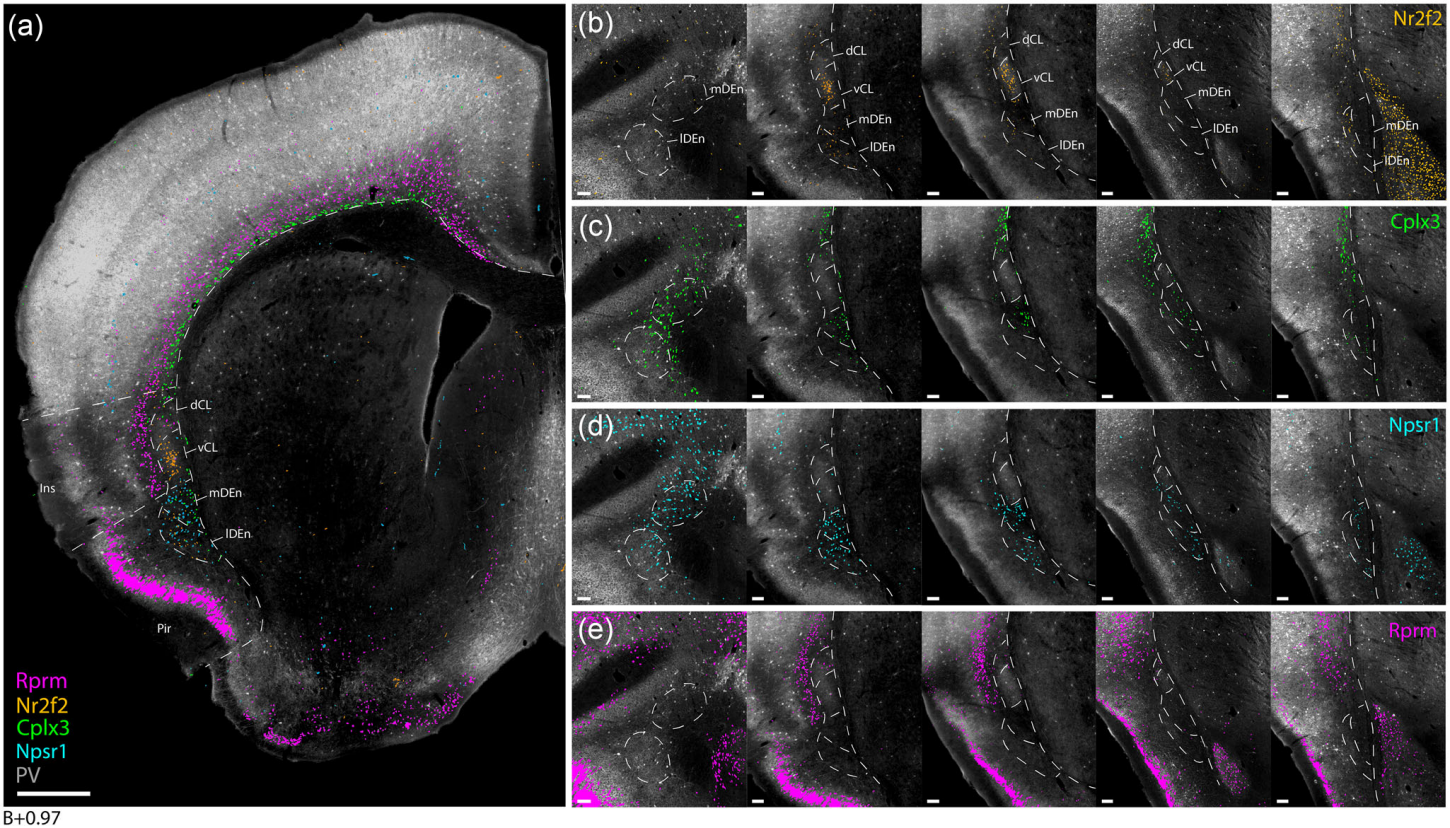
Genetic marker expression in the claustrum complex. Gene expression images were collected from the Allen hybridization (ISH) database and aligned to a reference section from a female C57 mouse (see Section 2 for details). (a) Combined expression of *Nr2f2*, *Cplx3*, *Npsr1*, and *Rprm* genes superimposed onto a reference section located at the rostral landmark. (b–e) Individual gene expression in the claustrum region from the five levels taken at the previously defined landmarks (see Figure 3), showing higher subregional specificity than more general markers like *Gnb4* and *Ntng2* (see Figure S2). Scale bars measure 500 μm in (a), and 100 μm in (b–e).

As several genes showed similar distribution patterns, we selected a few to analyze in more detail (Figure 4a). For these genes, we downloaded images of coronal slices at the five rostrocaudal landmarks and aligned them to a reference brain section using the image warping pipeline described in the methods (Figure 4b–e). We did not delineate the CLCX during the image warping procedure but used well‐defined borders of surrounding areas like the external capsule and borders among insular, piriform, and SSc. Delineations were drawn on the reference section, which was labeled with MBP, PV, and Cresyl Violet, without viewing the gene expression. PV‐labeling was selected for the background as this provided the clearest visual distinction to the superimposed data.

We found similar expression patterns among some of the genetic markers. Genes, such as *Nr2f2* and *Rxfp1*, displayed a dense, centered expression within vCL, in addition to sparser labeling in lDEn (Figure 4b), and genes like *Cplx3*, *Ctgf*, and *Galnt10* showed confined expression along the cortical subplate that stopped upon reaching the CL but reappeared in mDEn (Figure 4c). The *Npsr1* gene had dense and highly specific expression in both mDEn and lDEn (Figure 4d). Among layer 6 markers, the *Rprm* gene stood out with a dense laminar expression throughout L6 of the entire neocortex, and laterally along the outside of the CLCX (Figure 4e). The expressions of *Nr2f2*, *Cplx3*, and *Npsr1* align with fiber‐architectural delineations of the CLCX, and the expression of the *Rprm* gene was clearly aligned with the area lacking PV labeling, delineating layer 6 of insular cortex. Note that prior studies have characterized the expression of *Npsr1*, *Nr2f2*, and *Cplx3* in the CLCX (Bruguier et al., 2020; Clark et al., 2011; Erwin et al., 2021; Hoerder‐Suabedissen et al., 2023; Wang et al., 2017), but not in comparison to a fiber‐architectural reference. To our knowledge, the expression of the *Rprm* gene has not been characterized before for the CLCX. We did not see any markers showing specific expression in the dCL, although markers with a general expression in the entire CLCX, such as *Gnb4* and *Ntng2*, also include expression in the dCL (Figure S2).

### 3.3 Fiber‐architectural location of the retrosplenial‐projecting claustrum pocket

**FIGURE 5:**
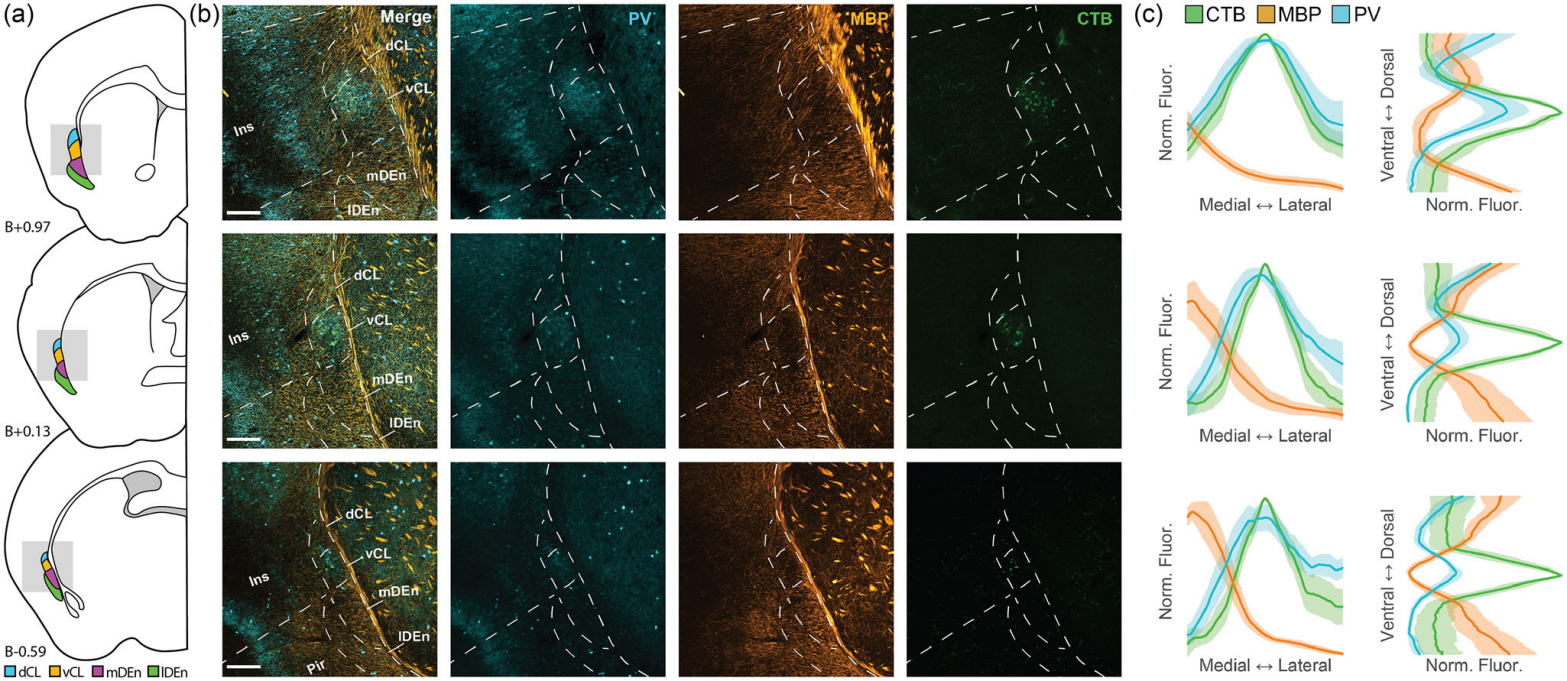
Retrosplenial cortex (RSC)‐projecting claustrum neurons overlap with the parvalbumin (PV) peak and the myelin basic protein (MBP) trough in the CL of a male C57 mouse. (a) Schematic representation of tissue sections used for immunohistochemical (IHC) and further analysis. (b) Representative images of the claustrum complex (CLCX) from sections depicted in (a) with retrogradely labeled claustrum neurons (green) and immunostaining against MBP (orange) and PV (cyan). Scale bars represent 200 μm. Images show maximum intensity projection of confocal images. (c) Normalized fluorescence traces for each of the sections in (b) along the mediolateral (left) and dorsoventral (right) axes. Shaded areas represent 95% confidence intervals.

A dense pocket of cells can be labeled in the CL by injecting a retrograde tracer into the RSC (Zingg et al., 2018). To assess the location of this RSC‐projecting pocket of CL cells (CL_RSC_‐pocket), relative to our fiber‐architectural markers, we injected CTB into the RSC of C57BL6J mice (*n* = 6). The tissue was then stained for the expression of MBP and PV (Figure 5a,b). The dense CL_RSC_‐pocket of labeled neurons shows a strong overlap with the MBP‐gap, though a more dispersed population of cells was seen in surrounding parts of the CL. Fluorescence profiles, measured along the mediolateral and dorsoventral axes through the center of the CL_RSC_‐pocket, showed a good alignment of the labeled neurons along both axes with the peak of PV fluorescence and trough of MBP fluorescence (Figure 5c). Note that at rostral levels the MBP trough was shifted slightly ventral to the CL_RSC_‐pocket.

### 3.4 Brain‐wide monosynaptic inputs to the mouse CLCX

**FIGURE 6:**
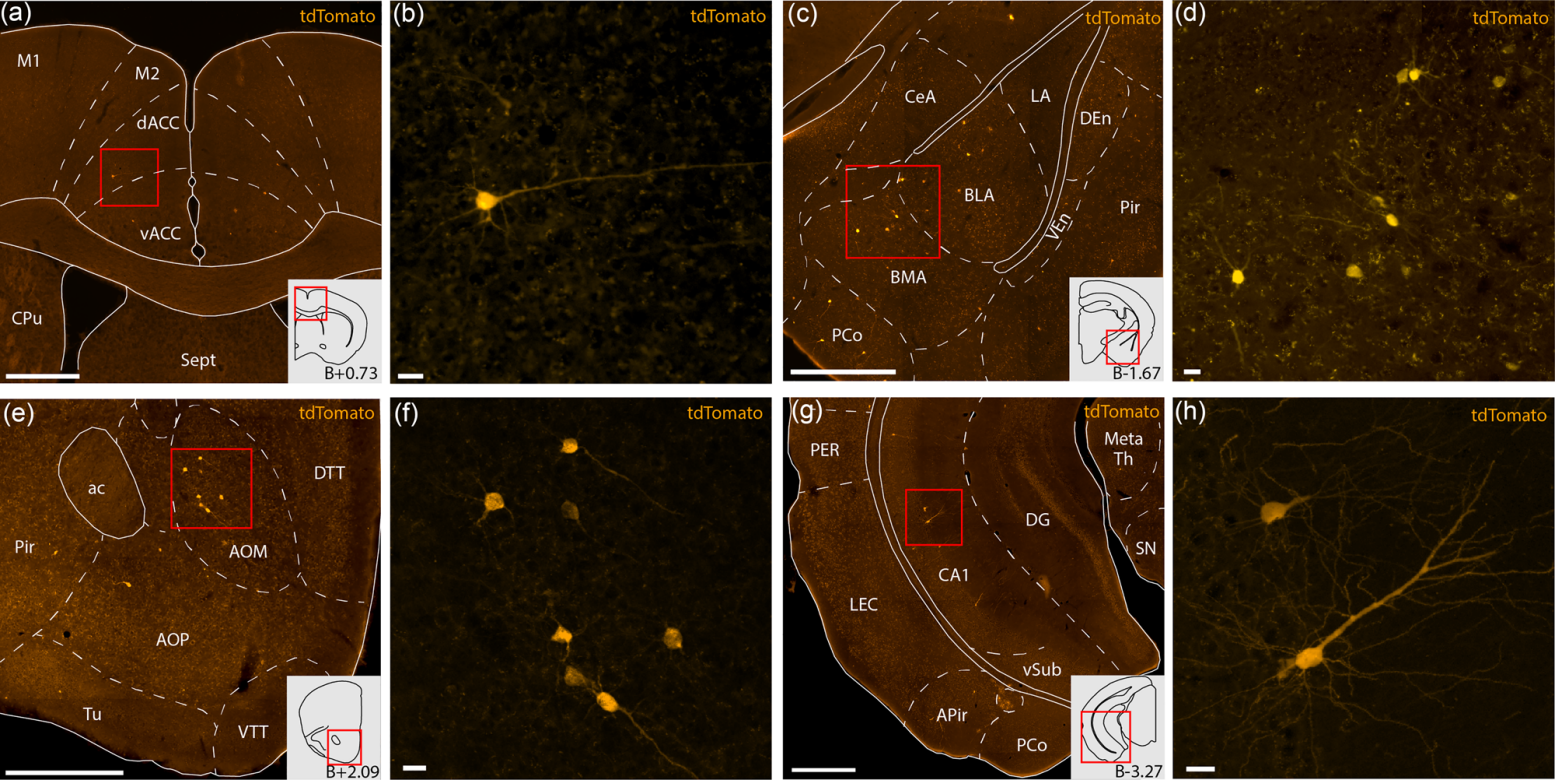
Example sections from rabies tracing dataset showcasing monosynaptic input cells projecting to the claustrum complex (CLCX) from anterior cingulate cortex (ACC; a and b), basolateral amygdala (BLA; c and d), medial anterior olfactory area (AOM; e and f), and *cornu ammonis* 1 (CA1; g and h). All delineations are based on Cresyl Violet staining of the same tissue. Input cells are labeled by a tdTomato‐tag expressed by the rabies virus. Schematics in the bottom right corner of panels a, d, e, and g show outlines of each representative coronal section, and the approximate distance relative to bregma. Scale bars measure 500 μm in panels a, c, e, g and 20 μm in panels b, d, f and h. Abbreviated terms are explained in the list of abbreviations. Images were taken with a slide scanner and shows sections from two female animals Rb11 (a and b) and Rb2 (c and d), and two male animals Rb10 (e and f) and Rb12 (g and h).

Using the CLCX‐EDGE::Tre‐Tight‐THAG transgenic mouse line, we conducted monosynaptic rabies‐tracing of brain‐wide inputs to all subregions of the CLCX. The location of each input and starter cell was determined based on Cresyl Violet staining. As such, the precise location of every cell could be determined within each animal. Input cells were found in a myriad of cortical and subcortical areas, expanding the input connectivity known from the literature (Figure 6). Considerable inputs were identified coming from known input areas like the ACC (Figure 6a,b), the amygdala (Figure 6c,d) and anterior olfactory areas (Figure 6e,f), and from less documented input areas like the hippocampal formation (Figure 6g,h).

**FIGURE 7:**
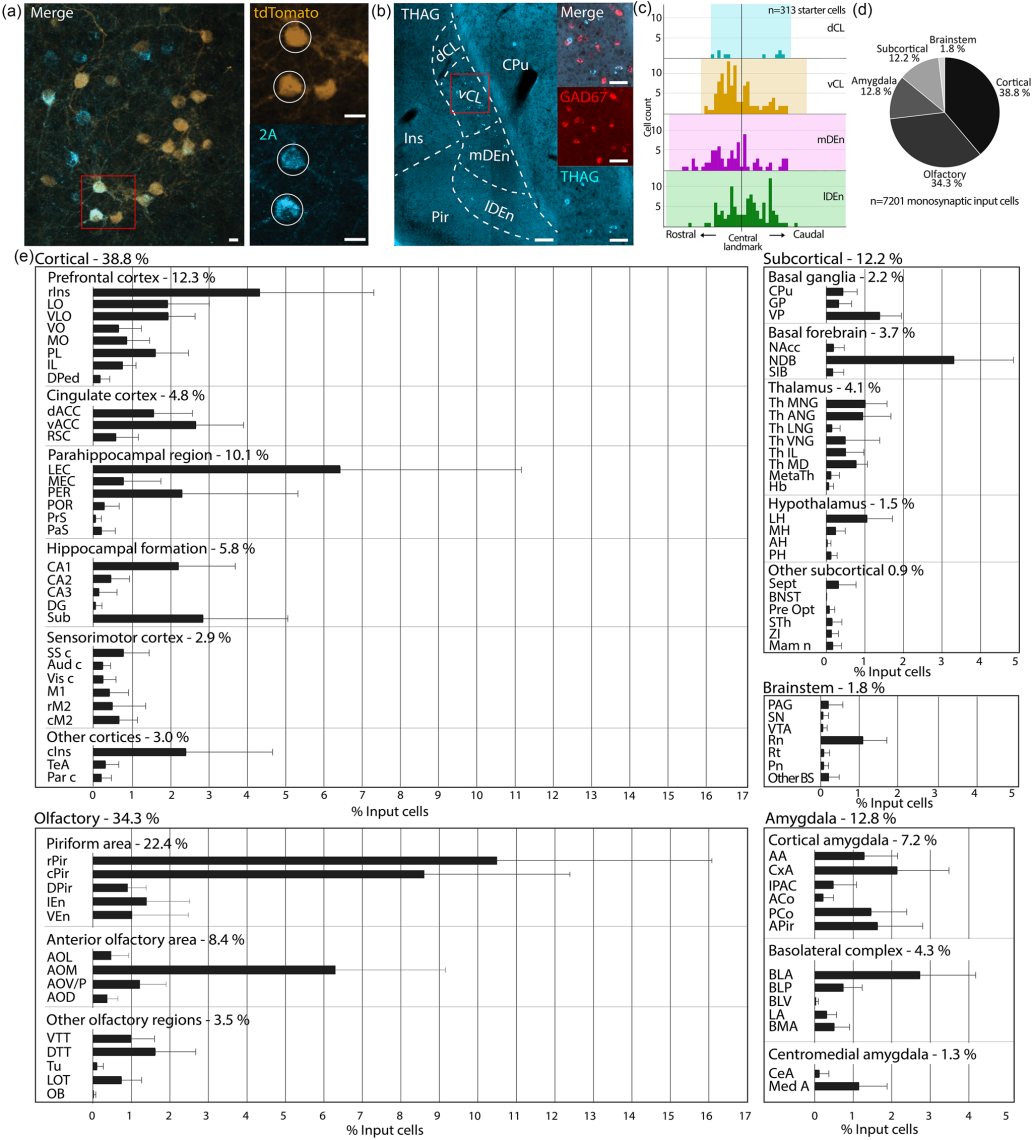
Brain‐wide monosynaptic input tracing to the claustrum complex (CLCX). (a) Confocal image (maximum intensity projection) of mouse Rb10 showing starter cells in the CL, identified by the co‐expression of tdTomato, expressed by the rabies virus, and the 2A linker protein, expressed in transgene expressing cells. Scale bars measure 20 μm. (b) Double in situ hybridization against THAG sequence, found in transgene expressing cells, and the general inhibitory marker GAD67. The image is taken with a slide scanner and is of a male CLCX‐EDGE::Tre‐Tight‐THAG animal. (c) Histograms showing rostrocaudal position of pooled starter cells from all animals within claustral subregions. (d) Coarse overview of extrinsic monosynaptic inputs to the CLCX. (e) Detailed overview of extrinsic monosynaptic inputs to the CLCX (*n* = 7201 cells, error bars show the standard error of the mean). For additional quantification, see Figure S3. Abbreviated terms are explained in the list of abbreviations.

Starter cells were IHC identified by the co‐expression of the 2A linker protein and a tdTomato tag (Figure 7a); input cells were identified by the expression of tdTomato, but not the 2A linker protein. We observed no co‐expression in double in situ staining against the THAG sequence, found in transgene expressing cells of the CLCX‐EDGE::Tre‐Tight‐THAG line, and GAD67, a general inhibitory marker, indicating that the starter cells were excitatory neurons (Figure 7b). Across 13 animals, 95.8% (±1.5% SE) of all starter cells were located within the CLCX; a few were found in nearby cortices (Table 6). Within the CLCX, starter cells were found in each dorsoventral subregion, although the dCL only contained a minority (Figure 7c). The starter cell population covered most of the rostrocaudal extent of the CLCX, the exception being far caudal parts of the mDEn and lDEn.

**TABLE 6:** Starter cell distributions.

Region	Starter cells per region in animals 1–13												
	Rb1	Rb2	Rb3	Rb4	Rb5	Rb6	Rb7	Rb8	Rb9	Rb10	Rb11	Rb12	Rb13
dCL	0	1	1	1	1	2	2	0	0	0	1	1	0
vCL	2	11	4	5	12	12	2	2	9	8	29	7	7
mDEn	2	9	2	4	0	5	2	5	8	6	27	6	0
lDEn	3	12	6	7	5	7	2	9	20	12	18	1	0
Other	1	0	1	0	0	4	0	0	5	2	2	0	0
Tot	8	33	14	17	18	30	8	16	42	28	77	15	7

We divided the extrinsic input connectivity into five major categories (Figure 7d): cortical (38.8 ± 3.6% SE), subcortical (12.2 ± 0.7% SE), olfactory (34.3 ± 3.4% SE), amygdala (12.8 ± 1.3% SE), and brainstem (1.8 ± 0.3% SE). In total, we found afferent neurons in 89 cytoarchitecturally defined regions (Figure 7e). This included known input areas to the CL like the prefrontal cortex, thalamic nuclei, and the basolateral amygdala. Known inputs to the DEn like anterior olfactory areas, cortical amygdala and LEC were also prevalent in the dataset. Additionally, we found considerable inputs from hippocampal regions, mainly in *cornu ammonis* 1 (CA1) and the subiculum. There were also cells in CA2, CA3 and the dentate gyrus, which have not been described before. Other as yet undescribed input regions include the habenula, bed nucleus of the stria terminalis and zona incerta (Table S1).

A small fraction of input cells was found in the contralateral hemisphere, and these cells were largely present in prefrontal and cingulate areas (Table S2). Some regions like the anterior cingulate (ACC) and prelimbic cortex, showed a balanced distribution of ipsi‐ and contralateral inputs, whereas areas like the basolateral amygdala and LEC showed a skewed distribution with only a small fraction of contralateral inputs. Notably, we observed contralateral inputs from the nucleus of the diagonal band, lateral hypothalamus, and the nucleus of the lateral olfactory tract, which have not been previously shown to project bilaterally to the CLCX. Areas along the midline of the brain were not categorized as ipsi‐ or contralateral. The number of input cells per animal was typically between 100 and 500 cells, with two outliers showing more than 1000 input cells. No clear trend was found between number of input cells and injection volume or number of starter cells, and there was no apparent difference between male and female animals (Figure S3).

**FIGURE 8:**
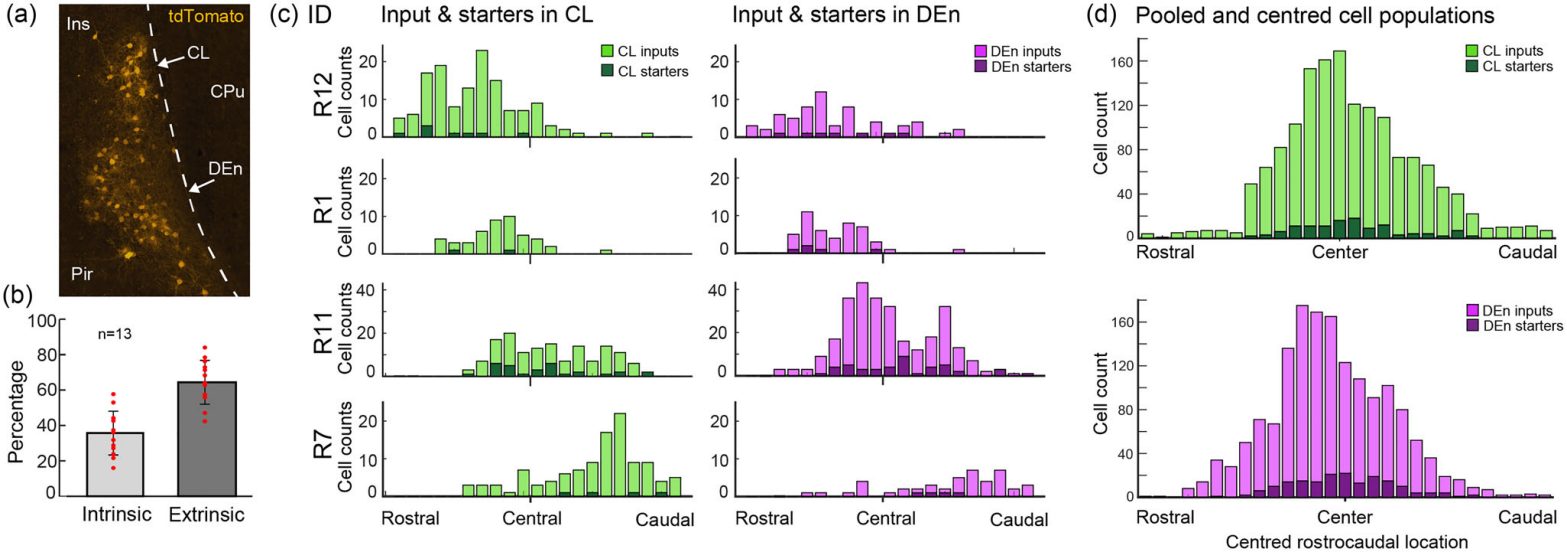
Intrinsic connectivity of the claustrum complex (CLCX). (a) Example brain section showing a dense population of intrinsic inputs in the CLCX of animal R11. (b) Comparison of intrinsic and extrinsic inputs across animals. (c) Distribution of input and starter cells from rostral to caudal sections in representative cases. Each distribution is anchored to where the anterior commissure joins at the midline (i.e., the central landmark for the CLCX). (d) Pooled input and starter populations aligned to the median section of each starter population.

In addition to the extrinsic inputs, input cells were also highly abundant within the CLCX (Figure 8a), comprising 37.5% on average of the total number of inputs across animals (Figure 8b). Intrinsic inputs were found in all CLCX subregions, along the rostrocaudal extent of the CLCX (Figure 8c). To study the rostrocaudal spread of inputs relative to their respective starter population, we aligned the median position of each starter population and visualized the pooled distribution of input cells across animals (Figure 8d). Although we saw, in both CL and DEn, that input populations were largely found in sections containing starter cells, we also observed substantial inputs in sections without starter cells, indicating an extensive intrinsic longitudinal connectivity.

**FIGURE 9:**
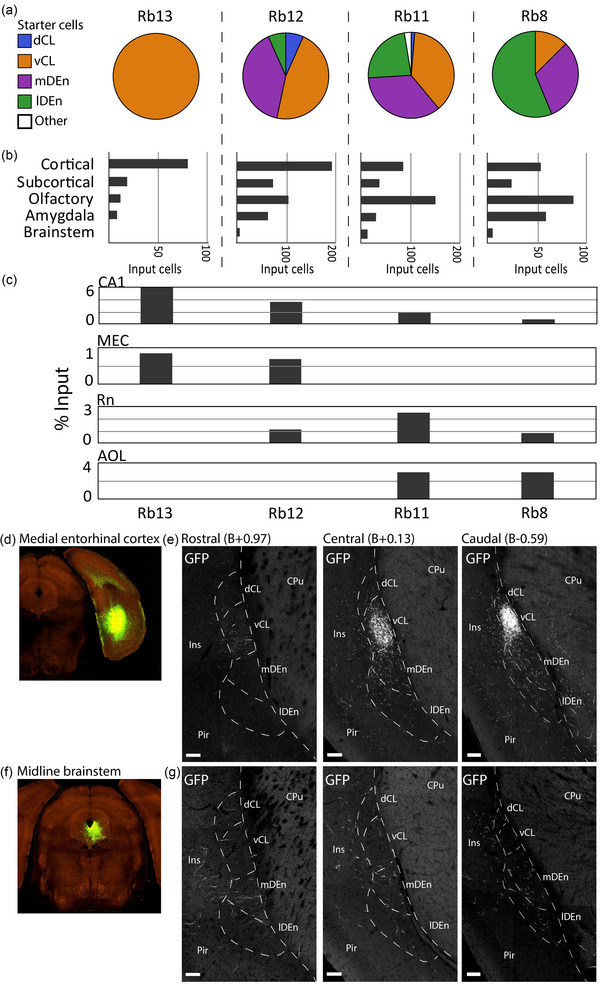
Comparison of input and starter cell populations. (a) Starter cell distributions in four representative brains displaying various proportions of starter cells in the claustrum complex (CLCX). (b) Coarse input distributions in the same four brains. (c) Percentage of inputs from *cornu ammonis* 1 (CA1), medial entorhinal cortex (MEC), raphe nuclei (Rn), and lateral anterior olfactory nucleus (AOL), in representative brains. (d) Injection site for experiment 518745840 with an injection of rAAV‐EGFP anterograde tracer in the MEC (2017 Allen Institute for Brain Science, Projection Dataset, Available from: https://connectivity.brain‐map.org/). (e) Axonal projections from MEC innervating rostral, central, and caudal parts of the claustrum complex. Images were warped onto matching reference‐sections to align the signal with fiber‐ and cytoarchitectural delineations. (f) The same as d, but from experiment 272699357 with the same anterograde tracer deposited in midline brainstem areas Rn and periaqueductal gray. (g) The same as in e, but from experiment 272699357. Scale bars measure 100 μm. Image credit (d–g): Allen Institute.

We selected a few representative brains with decreasing proportions of starter cells within the CL (Figure 9a). Among these, the ones with more CL‐starter cells had a higher representation of cortical inputs, and less olfactory and brainstem inputs (Figure 9b). Inputs from MEC and CA1 were more prevalent in brains with a high percentage of CL starter cells, while raphe nuclei (Rn) and lateral anterior olfactory area (AOL) inputs showed an opposite trend (Figure 9c). Using the Allen Projection database, we found a corresponding pattern in afferent projections from MEC and midline regions of the brainstem (Table 7). A coarse measure of axonal density was scored based on the density scale shown Figure S4. Images collected from these databases were fitted to our reference brain using image warping, allowing the signal to be superimposed onto sections delineated based on fiber‐ and cytoarchitecture. Corroborating the input patterns seen in our dataset, the MEC injected brains showed dense labeling in the vCL (Figure 9d), while injections in the midline of the brainstem showed labeling predominantly located in the mDEn, and surrounding the vCL, (Figure 9e). These labeling patterns thus align with the architecturally delineated vCL and mDEn.

**TABLE 7:** List of experiments collected from the Allen projection database.

Experiment	Injection site	Approximate fiber density				Mouse line
		dCL	vCL	mDEn	lDEn	
**Midline brainstem**						
310176384	Rn, Pn	–	–	++	+	Calb2‐IRES‐Cre
301732962	Rn, Pn, PAG	–	+	++	+	Slc18a2‐Cre_OZ14
301765327	Rn, PAG	–	–	+	–	Calb2‐IRES‐Cre
183562831	Rn, PAG	–	–	+	+	Cck‐IRES‐Cre
272699357	Rn, PAG	+	+	++	+	Th‐Cre_FI172
**Medial entorhinal cortex (MEC)**						
518745840	MEC	+	++++	+	+	Grp‐Cre_KH288
557199437	MEC	+	+++	+	+	Ntng2‐IRES2‐Cre
286484879	MEC, PrS	+	++	+	+	Syt17‐Cre_NO14

*Note*: ++++: extensive labeling; +++: dense labeling; ++: some labeling; +: sparse labeling;—: no labeling. Approximate fiber density was scored as shown Figure S4.

## 4 DISCUSSION

Borders for the rodent CLCX are difficult to define, and the use of various methods to do so has resulted in substantial variation among current delineation schemes (Bruguier et al., 2020; Dillingham et al., 2019; Fang et al., 2021; Smith et al., 2019; Wang et al., 2023). We combined a variety of strategies, several of which have been used previously, in search of overlapping patterns to delineate the CLCX as well as to define its constituting subdivisions. As a result, we present a multifaceted definition for the borders of the CLCX, based on expression patterns of multiple, methodologically different markers, and aided by established anatomical features of adjacent cortices like PV‐labeled neuropil in L5 of insular cortex and CB positive cells in L5‐6 of neocortex (Alcantara et al., 1993; Hof et al., 1999; Tremblay et al., 2016). Our results indicated that there are four distinct domains within the CLCX, which is in‐line with prior studies where both the CL and DEn have been divided into two (Binks et al., 2019; Fang et al., 2021; Smith et al., 2019), although some variation exists between our border placements and those previously proposed.

We present a highly detailed characterization of myelinated fiber‐patterns in the mouse CLCX. In general, MBP staining in the CLCX displayed intricate patterns that were highly useful for delineation. We observed clear differences in the amount of myelination within subregions of the CLCX, which is also seen in marmosets, where the CL is more myelinated than the DEn (Pham et al., 2019). Our motivation for studying MBP‐labeling was also due to the evolutionarily preserved fiber‐tracts encapsulating the CLCX in mammals (Bruguier et al., 2020; Buchanan & Johnson, 2011; Kowianski et al., 1999; Pham et al., 2019). As indicated by MBP staining lateral to the CL, an extreme‐capsule‐equivalent could exist in mice, albeit merged into L5‐6 of insular cortex. It would be interesting to see if similar patterns exist in other animals lacking a clear extreme capsule, like rats, fruit bats, or pangolins.

Our research also contains novel characterizations of PV and CB expression in the CLCX. We discovered a PV‐plexus in the lDEn which has not been described in prior studies (Druga et al., 1993; Real et al., 2003; Suzuki & Bekkers, 2010). Additionally, we characterized a ringlike CB‐plexus in the CL, which has not been identified before, although sparsity of CB‐labeling has been described (Celio, 1990; Davila et al., 2005; Druga et al., 1993). The CB‐plexus was also visible in the CL anterior to the striatum, corroborating borders indicated by *Crym* ISH‐labeling (Dillingham et al., 2017). The unique appearance of the CB‐plexus, which reliably follows the PV‐plexus in the CL also at the rostral extremes, makes a solid argument for the existence of a CL‐domain anterior to the striatum. Earlier work has placed the rostral‐most borders for the CL ventrolateral to the forceps minor (Grasby & Talk, 2013; Jankowski & O'Mara, 2015), but this area likely belongs to the cortex as it lacks a clear PV‐plexus and genetic markers for the CL (Mathur et al., 2009).

Since CB is expressed both in excitatory and inhibitory cell‐types (DeFelipe, 1997; Gonchar & Burkhalter, 1997), it is difficult to make functional assumptions about the CB‐plexus in the CL. However, there is a similarity between the CB‐plexus and SST labeling in the CL, which labels a major subtype of interneurons (Graf et al., 2020; Tremblay et al., 2016). Considering that some CB‐labeled cells co‐express PV, it could be that the CB‐plexus represents an inhibitory network in the CL. Fibers expressing CR, a marker that is mainly present in inhibitory neurons and rarely co‐expresses with CB, occupy the perimeter of the CL in a similar way to the CB‐plexus (Barinka & Druga, 2010; Davila et al., 2005; Real et al., 2003). Together, these networks of CB‐, CR‐, and SST‐positive neurites could represent an “inhibitory surround” that regulates the central pocket of excitatory projection neurons in the CL.

**FIGURE 10:**
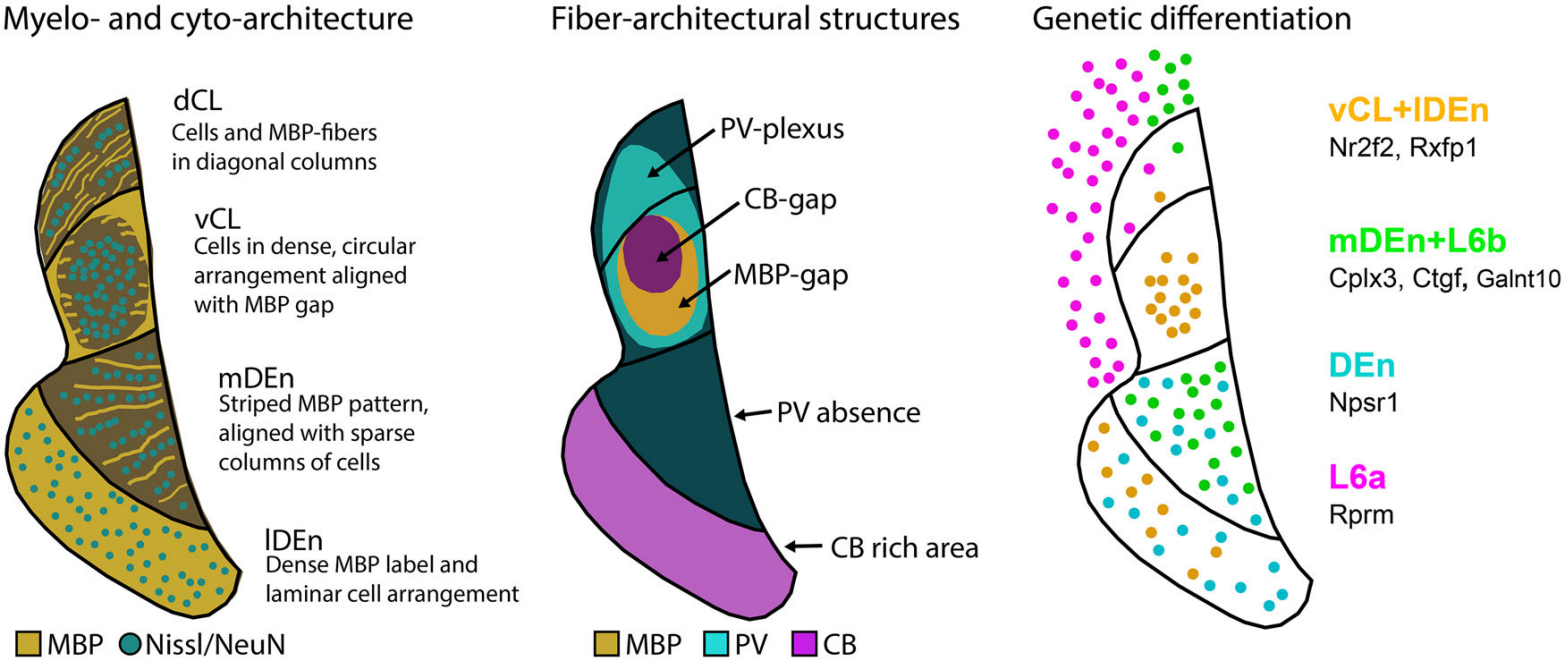
Schematic summary of main fiber‐ and cytoarchitectural, immunohistochemical, and genetic identifying features, proposed to provide standardized criteria to define and delineate the claustrum complex (CLCX) and its individual subdivisions. As can be seen, the dCL is genetically different from the three remaining subregions and can be delineated by the presence of diagonally oriented myelin basic protein (MBP) fibers and cellular architecture. In contrast, vCl has a dense expression of genetic markers like *Nr2f2* and *Rxfp1* and can be identified in experimental material by the unique clustering of cell bodies in an area that lacks MBP and calbindin (CB) labeling. The mDEn is uniquely labeled by markers that co‐express in the cortical subplate, like *Cplx3*, *Ctgf*, and *Galnt10*, and shows a striped pattern in both MBP labeling and cellular organization in addition to a complete absence of parvalbumin (PV) labeling. Lastly, the lDEn is genetically populated by the same markers that express in the vCL in addition to the *Npsr1* gene and can be delineated by a dense population of CB labeled cells or a sparser PV plexus, and by its dense cell packing relative to the mDEn and piriform cortex.

Divisional schemes for the CL show two overarching types: They either focus on differentiating between dorsoventral patterns (dCL and vCL) or on a center‐surround organization (core and shell). Our data indicate that these two organizational principles coexist (Figure 10), in‐line with what is proposed by Marriott et al. (2021). We here provide a unifying framework to anchor experimental data to generally applicable architectonic criteria (https://osf.io/sdwrv/). The transition from insular to piriform cortex has traditionally been considered to indicate the border between the CL and DEn, which our results show as well and further provide better foundation for this border. Note that we did not consider the intermediate and ventral endopiriform nuclei to be part of the CLCX, due to ontogenetic differences (Watson & Puelles, 2017). These regions were therefore not characterized. With our multifaceted delineation strategy, a crucial element of which is using the co‐expression of multiple markers in the same tissue, we discovered that the MBP‐gap only occupied a ventral part of the claustral PV‐plexus. This forms an argument for subdividing the CL into a dorsal and ventral domain and thus indicates that using the MBP‐gap to define claustral borders (Wang et al., 2023) might miss a dorsal part of what by others is considered the CL.

Gene expression gives insight into the genetic diversity of brain regions but only rarely does a gene express selectively in one area (Lein et al., 2007). For the CLCX, some of the claimed markers are also expressed in the adjacent insular cortex (Wang et al., 2017), which we observed as well. This is likely associated with the shared developmental origin of these structures from the lateral pallium. Cells destined for the CL and DEn migrate alongside those going to the insular cortex, and although the former class of neurons is largely distinguishable by the genetic marker *Nurr1* (Watson & Puelles, 2017), and *Nurr1* is indeed enriched in the CLCX, it is also expressed in the insular cortex (Arimatsu et al., 2003; Binks et al., 2019; Fang et al., 2021). The use of *Nurr1* to differentiate between cortex and CL therefore warrants some caution. Similarly, subdivisional schemes for the DEn, if delineated solely based on *Nurr1*‐expression (Fang et al., 2021), do not capture the clear mediolateral border we observed in multiple fiber‐architectural markers, which highlights the importance of our multifaceted delineation strategy.

Among the myriad of markers screened in this study, we observed two prevalent expression patterns in the CLCX. Genes like *Nr2f2* labeled the vCL and lDEn, and genes like *Cplx3* labeled the mDEn. This corroborates a genetic diversity previously described in the literature (Erwin et al., 2021; Watson & Puelles, 2017), and together with the unique features seen in our own data, distinguishes the mDEn area from other parts of the CLCX. Interestingly, projections from brainstem areas like the dorsal Rn and the periaqueductal gray selectively innervate the mDEn. These regions comprise a substantial fraction of subcortical inputs in our dataset and could be pertinent to the functional properties of the mDEn. Finally, our claustrocortical border, primarily defined by the PV‐plexus, coincided with the expression of the L6 marker *Rprm*.

As part of our characterization, we also conducted a comprehensive tracing study of brain‐wide inputs to the CLCX, using the CLCX‐EDGE transgenic mouse line (Blankvoort et al., 2018). An important aspect of our dataset is that the locations of all cells were anchored to cyto‐architectonically defined brain areas, instead of mapping them to atlas delineations. In total, we categorized inputs from 89 different brain regions, which considerably expands previous tracing studies targeting the CLCX. Further, we report brain‐wide inputs to the DEn, which is new to the field. We found substantial inputs arising in the CA‐regions of the hippocampus, which is a largely unexplored projection only documented in a few other studies (Wang et al., 2023; Zingg et al., 2018). Interestingly, CA1 inputs were not seen when rabies tracing was used with the Erg2‐transgenic mouse line (Atlan et al., 2018), which could indicate that only a subpopulation of CL cells receive inputs from these regions. We also showed that inputs intrinsic to the CLCX were highly abundant, corroborating recent findings on extensive rostrocaudal intraclaustral connectivity (Shelton et al., 2022).

## 5 CONCLUSION

Although the true complexity of the CLCX might be best described by gradients rather than by defining clear borders (Atlan et al., 2017; Marriott et al., 2021; Olson & Graybiel, 1980), simple delineations still hold a practical value. We present a robust delineation system, based on a few easy‐to‐use chemical markers that might serve the communication and comparison of data within the community. Ultimately, our descriptive strategies should be determined by the anatomical precision needed to support particular experimental claims. In some cases, it will suffice to retrogradely label the CL_RSC_‐pocket, whereas other experiments will call for multiple fiber‐ or cytoarchitectural markers to be expressed. We added a description of specific gene expression patterns aligned to anatomical features of both fiber‐ and cytoarchitectural markers, and we trust that this combinatorial approach provides a common referencing system to anchor data in future experiments on the functional organization of the CLCX.

## CONFLICT OF INTEREST STATEMENT

The authors declare no conflicts of interest.

### PEER REVIEW

The peer review history for this article is available at https://publons.com/publon/10.1002/cne.25539.

## Supporting information

Figure S1: MBP labeling from the same sections shown in Figure 2.
Figure S2: Superimposed ISH data showing genetic markers Gnb4 and Ntng2, which expresses generally in the claustrum complex as well as in nearby cortex.
Figure S3: Rabies tracing additional quantification.
Figure S4: Axon labeling scale showing examples of fiber densities that correspond to the grading in Table 7.

   

   

   

   

Table S1: Rabies tracing of monosynaptic (extrinsic) inputs to the claustrum complex.
Table S2: Contralateral inputs in rabies tracing dataset.

## Data Availability

The data that support the findings of this study are openly available in Center for Open Science.
