# Supplementary material for: A multifaceted architectural framework of the mouse claustrum complex
Source: J Comp Neurol. 2023 Oct 2;531(17):1772–95. doi: 10.1002/cne.25539 (PMC10953385; doi:10.1002/cne.25539)
Supplement: Supplementary file 1 — Figure S1: MBP labeling from the same sections shown in Figure 2. Figure S2: Superimposed ISH data showing genetic markers Gnb4 and Ntng2, which expresses generally in the claustrum complex as well as in nearby cortex. Figure S3: Rabies tracing additional quantification. Figure S4: Axon labeling scale showing examples of fiber densities that correspond to the grading in Table 7. [file CNE-531-1772-s003.docx]

**Supplementary figure legends**

**Figure S2-1:** MBP labeling from the same sections shown in figure 2. Note that staining against MBP only indicates borders to the CL in the most posterior of the five sections. Insets show the same areas as depicted in figure 2 C-D. Scale bars measure 500μm in overview images and 100μm in insets. All images were obtained with a slide scanner (see methods). Abbreviated terms are explained in the list of abbreviations.

**Figure S4-1**: Superimposed ISH data showing genetic markers Gnb4 and Ntng2, which expresses generally in the claustrum complex as well as in nearby cortex. ISH data were acquired from the Allen ISH database experiments [74047771](https://mouse.brain-map.org/experiment/show/74047771) and [73615803](https://mouse.brain-map.org/experiment/show/73615803), and processed as described in the methods section.

**Figure S7-1**: Rabies tracing additional quantification. Total number of input cells are compared to injection volume **(A)** and total number of starter cells **(B)**. Each point represents one animal. No apparent difference was found between female (violet) and male (green) animals.

**Figure S9-1:** Axon labelling scale showing examples of fiber densities that correspond to the grading in table 7. Images show labelling in the CC in four experiments taken from the Allen Projection database, experiments [183562831](https://connectivity.brain-map.org/projection/experiment/siv/183562831?imageId=183563366&imageType=TWO_PHOTON,SEGMENTATION&initImage=TWO_PHOTON&x=15840&y=9643&z=2), [286484879](https://connectivity.brain-map.org/projection/experiment/siv/286484879?imageId=286485265&imageType=TWO_PHOTON,SEGMENTATION&initImage=TWO_PHOTON&x=25337&y=10589&z=3), [557199437](https://connectivity.brain-map.org/projection/experiment/siv/557199437?imageId=557199846&imageType=TWO_PHOTON,SEGMENTATION&initImage=TWO_PHOTON&x=26934&y=14156&z=3) and [518745840](https://connectivity.brain-map.org/projection/experiment/siv/518745840?imageId=518746249&imageType=TWO_PHOTON,SEGMENTATION&initImage=TWO_PHOTON&x=25350&y=11402&z=3).
