# Supplementary figures and images for: A multifaceted architectural framework of the mouse claustrum complex
Source: J Comp Neurol. 2023 Oct 2;531(17):1772–95. doi: 10.1002/cne.25539 (PMC10953385; doi:10.1002/cne.25539)

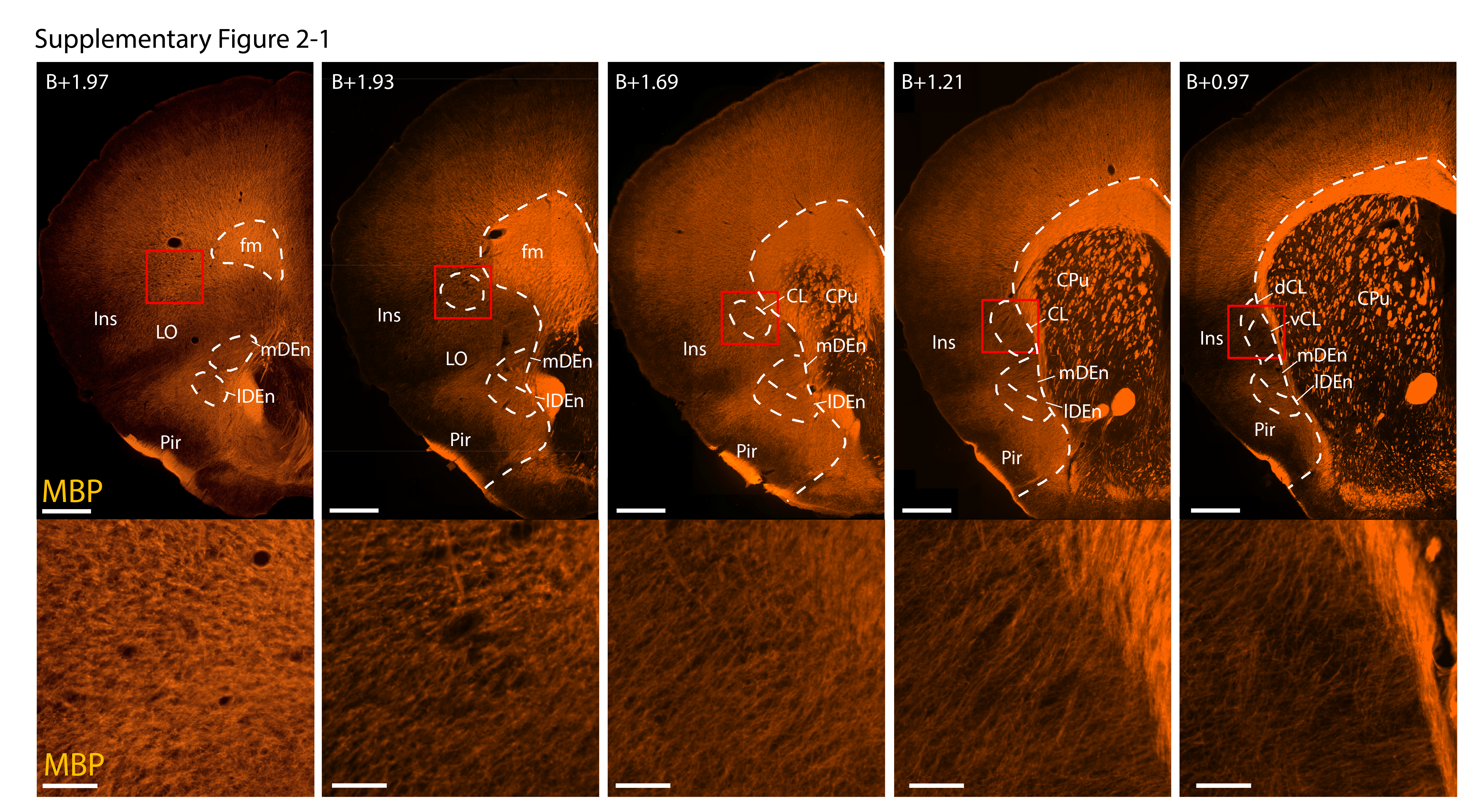

Supplement: Supplementary file 2 [file CNE-531-1772-s005.png]

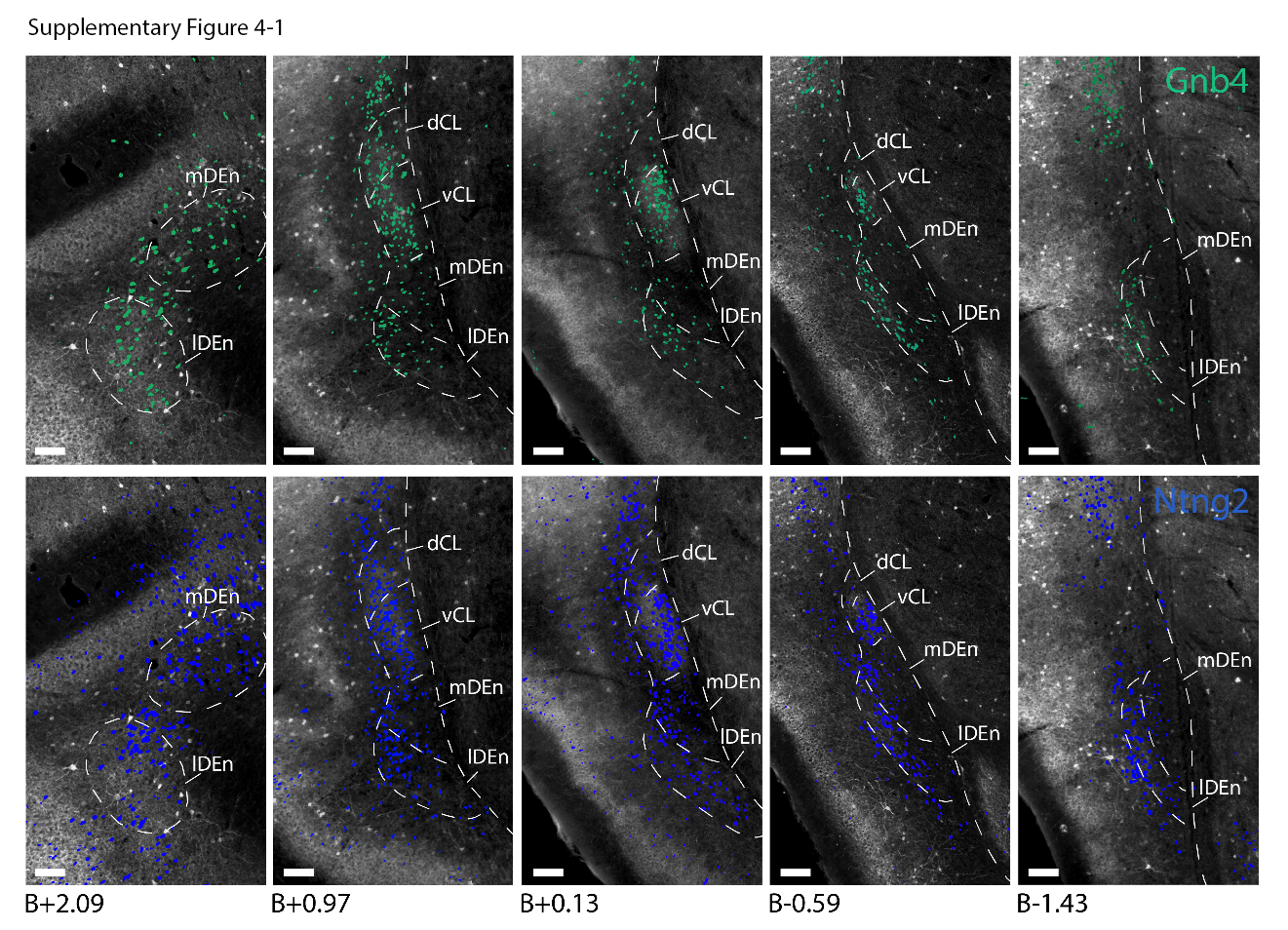

Supplement: Supplementary file 3 [file CNE-531-1772-s001.png]

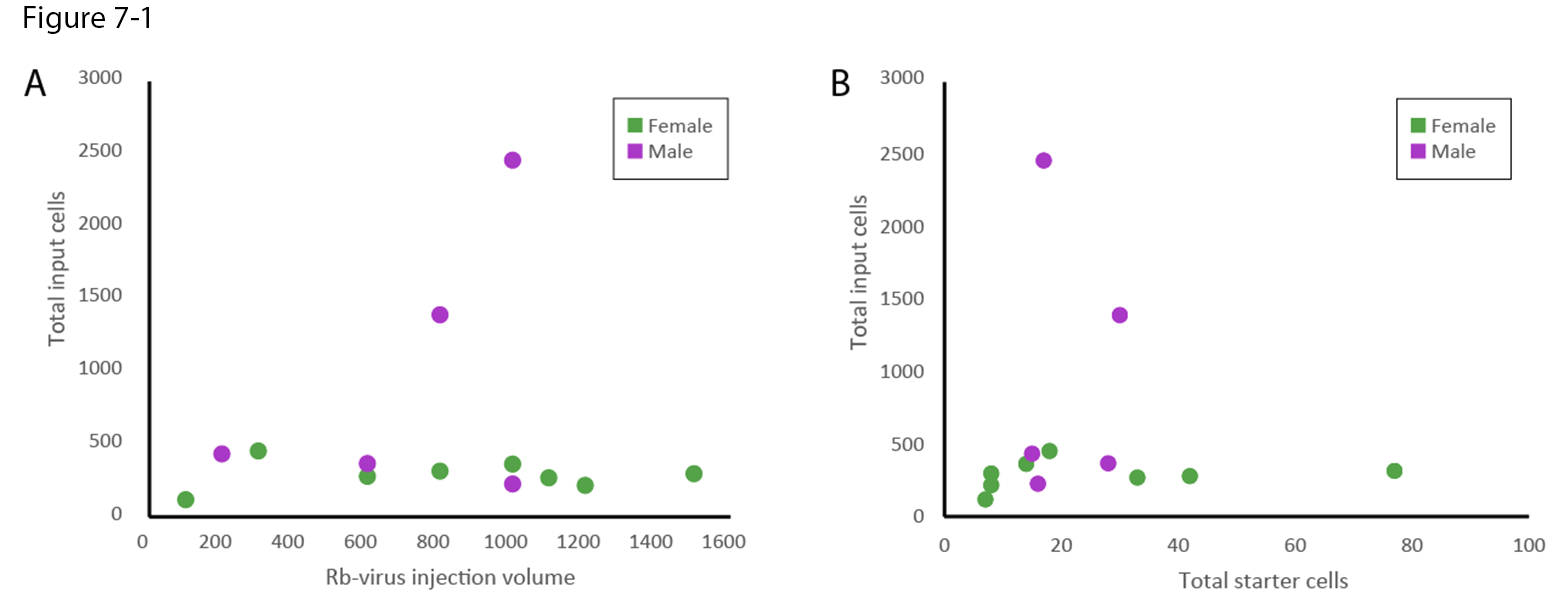

Supplement: Supplementary file 4 [file CNE-531-1772-s004.png]

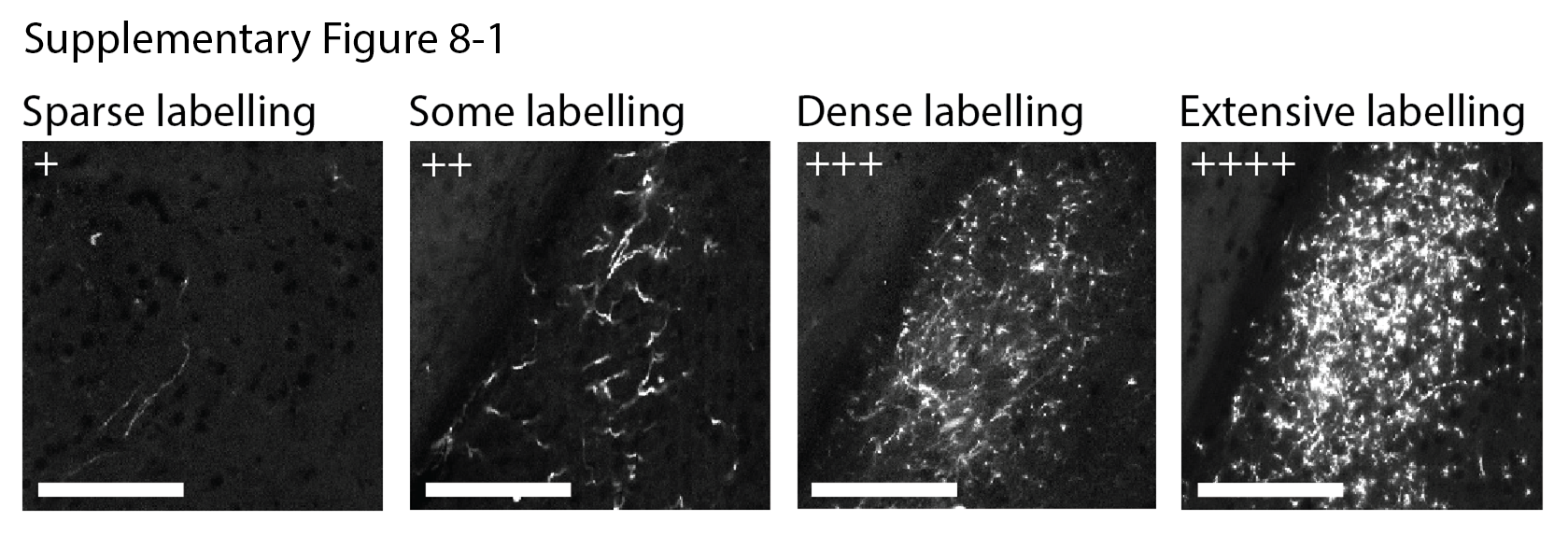

Supplement: Supplementary file 5 [file CNE-531-1772-s006.png]
