# Supplementary material for: A multifaceted architectural framework of the mouse claustrum complex
Source: J Comp Neurol. 2023 Oct 2;531(17):1772–95. doi: 10.1002/cne.25539 (PMC10953385; doi:10.1002/cne.25539)
Supplement: Supplementary file 6 — Table S1: Rabies tracing of monosynaptic (extrinsic) inputs to the claustrum complex. Table S2: Contralateral inputs in rabies tracing dataset. [file CNE-531-1772-s002.docx]

# **Supplementary data tables**

Table 7-1: Rabies tracing of monosynaptic (extrinsic) inputs to the claustrum complex

| Coarse categorizations | | Region | Input cells per region in animals Rb1-Rb13 | | | | | | | | | | | | |
| --- | --- | --- | --- | --- | --- | --- | --- | --- | --- | --- | --- | --- | --- | --- | --- |
|  |  |  | Rb1 | Rb2 | Rb3 | Rb4 | Rb5 | Rb6 | Rb7 | Rb8 | Rb9 | Rb10 | Rb11 | Rb12 | Rb13 |
| Cortical | Prefrontal cortex | rIns | 38 | 20 | 7 | 89 | 15 | 22 | 7 | 4 | 10 | 10 | 10 | 19 | 8 |
|  |  | LO | 9 | 4 | 7 | 47 | 1 | 45 | 1 | 2 | 6 | 5 | 7 | 9 | 5 |
|  |  | VLO | 9 | 4 | 5 | 63 | 8 | 28 | 5 | 1 | 6 | 9 | 3 | 10 | 3 |
|  |  | VO | 2 | 2 | 3 | 17 | 0 | 17 | 2 | 0 | 4 | 7 | 0 | 1 | 0 |
|  |  | MO | 1 | 0 | 1 | 43 | 4 | 14 | 4 | 1 | 1 | 6 | 2 | 6 | 1 |
|  |  | PL | 3 | 2 | 4 | 66 | 2 | 31 | 5 | 1 | 4 | 6 | 5 | 9 | 4 |
|  |  | IL | 3 | 1 | 2 | 31 | 4 | 10 | 2 | 1 | 4 | 1 | 1 | 4 | 1 |
|  |  | DPed | 0 | 2 | 0 | 1 | 1 | 2 | 0 | 0 | 1 | 1 | 2 | 0 | 0 |
|  | Cingulate cortex | dACC | 8 | 1 | 1 | 51 | 4 | 27 | 5 | 0 | 7 | 10 | 1 | 8 | 3 |
|  |  | vACC | 10 | 4 | 4 | 63 | 4 | 34 | 8 | 1 | 12 | 15 | 11 | 15 | 4 |
|  |  | RSC | 1 | 0 | 1 | 18 | 1 | 6 | 2 | 0 | 3 | 1 | 5 | 8 | 0 |
|  | Parahippocampal region | LEC | 9 | 9 | 17 | 136 | 87 | 83 | 19 | 12 | 13 | 4 | 14 | 17 | 16 |
|  |  | MEC | 2 | 1 | 1 | 37 | 3 | 15 | 0 | 0 | 1 | 14 | 0 | 3 | 1 |
|  |  | PER | 3 | 1 | 10 | 34 | 2 | 11 | 23 | 9 | 0 | 2 | 3 | 2 | 8 |
|  |  | POR | 2 | 0 | 0 | 4 | 1 | 3 | 3 | 0 | 0 | 1 | 2 | 1 | 0 |
|  |  | PrS | 0 | 0 | 0 | 1 | 0 | 0 | 1 | 0 | 1 | 0 | 0 | 0 | 0 |
|  |  | PaS | 0 | 0 | 1 | 1 | 0 | 3 | 2 | 0 | 0 | 4 | 1 | 0 | 0 |
|  | Hippocampal formation | CA1 | 11 | 1 | 5 | 36 | 12 | 40 | 3 | 2 | 5 | 3 | 6 | 16 | 7 |
|  |  | CA2 | 0 | 0 | 1 | 6 | 1 | 7 | 1 | 0 | 1 | 3 | 1 | 5 | 2 |
|  |  | CA3 | 0 | 0 | 0 | 43 | 0 | 0 | 0 | 0 | 0 | 0 | 0 | 1 | 0 |
|  |  | DG | 0 | 0 | 0 | 14 | 0 | 0 | 0 | 0 | 0 | 1 | 0 | 0 | 0 |
|  |  | Sub | 4 | 1 | 6 | 19 | 29 | 38 | 5 | 14 | 3 | 7 | 4 | 30 | 5 |
|  | Sensorimotor cortex | SSc | 3 | 1 | 2 | 20 | 1 | 9 | 4 | 0 | 2 | 2 | 1 | 3 | 3 |
|  |  | Aud c | 2 | 0 | 1 | 7 | 0 | 4 | 1 | 1 | 1 | 1 | 0 | 1 | 0 |
|  |  | Vis c | 0 | 0 | 0 | 5 | 1 | 8 | 2 | 0 | 0 | 1 | 3 | 1 | 0 |
|  |  | M1 | 0 | 2 | 0 | 27 | 1 | 4 | 0 | 0 | 4 | 0 | 0 | 4 | 1 |
|  |  | rM2 | 0 | 1 | 1 | 19 | 2 | 3 | 0 | 0 | 0 | 1 | 1 | 2 | 4 |
|  |  | cM2 | 3 | 0 | 0 | 24 | 3 | 10 | 3 | 1 | 3 | 3 | 1 | 6 | 0 |
|  | Other cortices | cIns | 4 | 7 | 12 | 14 | 11 | 12 | 20 | 4 | 10 | 1 | 2 | 6 | 4 |
|  |  | TeA | 1 | 0 | 1 | 10 | 0 | 2 | 2 | 0 | 1 | 3 | 0 | 4 | 0 |
|  |  | Par. c | 0 | 0 | 0 | 14 | 0 | 4 | 1 | 0 | 2 | 2 | 0 | 1 | 0 |
| Subcortical | Basal ganglia | CPu | 0 | 1 | 1 | 4 | 0 | 3 | 1 | 2 | 3 | 3 | 3 | 2 | 0 |
|  |  | GP | 3 | 0 | 0 | 14 | 3 | 5 | 0 | 1 | 0 | 2 | 2 | 0 | 0 |
|  |  | VP | 4 | 5 | 4 | 27 | 9 | 25 | 3 | 3 | 1 | 6 | 3 | 3 | 3 |
|  | Basal forebrain | NAcc | 0 | 1 | 0 | 3 | 0 | 3 | 0 | 0 | 0 | 2 | 1 | 4 | 0 |
|  |  | NDB | 10 | 9 | 22 | 77 | 23 | 39 | 1 | 8 | 1 | 14 | 15 | 17 | 3 |
|  |  | SIB | 2 | 0 | 2 | 5 | 0 | 11 | 0 | 0 | 0 | 0 | 0 | 0 | 0 |
|  | Thalamus | Th MNG | 2 | 1 | 4 | 60 | 4 | 14 | 1 | 3 | 3 | 2 | 2 | 8 | 1 |
|  |  | Th ANG | 0 | 2 | 1 | 21 | 4 | 9 | 1 | 0 | 5 | 5 | 3 | 8 | 3 |
|  |  | Th LNG | 1 | 0 | 1 | 5 | 3 | 0 | 0 | 1 | 0 | 0 | 0 | 0 | 0 |
|  |  | Th VNG | 0 | 1 | 0 | 13 | 0 | 5 | 0 | 0 | 3 | 1 | 0 | 2 | 4 |
|  |  | Th IL | 1 | 2 | 0 | 19 | 2 | 11 | 0 | 0 | 2 | 3 | 0 | 1 | 2 |
|  |  | Th MD | 3 | 2 | 0 | 14 | 5 | 8 | 2 | 2 | 3 | 2 | 3 | 4 | 1 |
|  |  | MetaTh | 0 | 0 | 0 | 1 | 0 | 1 | 0 | 1 | 1 | 0 | 0 | 3 | 0 |
|  |  | Hb | 0 | 0 | 0 | 0 | 0 | 0 | 0 | 0 | 1 | 1 | 0 | 1 | 0 |
|  | Hypothalamus | LH | 2 | 1 | 5 | 39 | 1 | 20 | 4 | 1 | 4 | 7 | 2 | 8 | 0 |
|  |  | MH | 1 | 0 | 0 | 8 | 0 | 5 | 1 | 0 | 1 | 0 | 2 | 3 | 0 |
|  |  | AH | 1 | 0 | 0 | 1 | 0 | 0 | 0 | 0 | 0 | 0 | 0 | 0 | 0 |
|  |  | PH | 1 | 0 | 0 | 5 | 0 | 2 | 1 | 0 | 0 | 1 | 0 | 1 | 0 |
|  | Other subcortical areas | Sept | 1 | 0 | 0 | 1 | 1 | 10 | 0 | 0 | 1 | 0 | 1 | 2 | 2 |
|  |  | BNST | 0 | 0 | 0 | 1 | 0 | 3 | 0 | 0 | 0 | 0 | 0 | 0 | 0 |
|  |  | Preoptc | 0 | 0 | 0 | 5 | 2 | 2 | 0 | 0 | 0 | 1 | 0 | 0 | 0 |
|  |  | STh | 0 | 0 | 0 | 2 | 1 | 6 | 1 | 0 | 0 | 3 | 0 | 0 | 0 |
|  |  | ZI | 0 | 0 | 0 | 1 | 0 | 3 | 1 | 1 | 1 | 1 | 0 | 0 | 0 |
|  |  | Mam. n | 1 | 1 | 0 | 6 | 0 | 2 | 0 | 1 | 0 | 0 | 0 | 3 | 0 |
| Olfactory | Piriform area | rPir | 51 | 51 | 49 | 143 | 35 | 148 | 5 | 20 | 43 | 45 | 52 | 33 | 0 |
|  |  | cPir | 19 | 33 | 36 | 167 | 68 | 133 | 9 | 18 | 38 | 36 | 35 | 13 | 3 |
|  |  | DPir | 5 | 4 | 5 | 23 | 1 | 11 | 2 | 3 | 3 | 2 | 3 | 2 | 0 |
|  |  | IEn | 4 | 11 | 5 | 22 | 5 | 15 | 2 | 0 | 3 | 10 | 9 | 3 | 0 |
|  |  | VEn | 0 | 15 | 1 | 7 | 0 | 9 | 1 | 5 | 3 | 6 | 2 | 2 | 0 |
|  | Anterior olfactory area | AOL | 0 | 0 | 4 | 15 | 1 | 7 | 0 | 3 | 2 | 3 | 3 | 0 | 0 |
|  |  | AOM | 8 | 24 | 40 | 266 | 10 | 59 | 7 | 18 | 11 | 19 | 23 | 34 | 8 |
|  |  | AOV/P | 2 | 7 | 7 | 29 | 2 | 22 | 0 | 4 | 3 | 3 | 6 | 5 | 1 |
|  |  | AOD | 0 | 2 | 1 | 19 | 2 | 2 | 0 | 1 | 2 | 1 | 2 | 2 | 0 |
|  | Other olfactory areas | VTT | 1 | 2 | 6 | 19 | 2 | 23 | 1 | 5 | 3 | 4 | 5 | 4 | 0 |
|  |  | DTT | 13 | 2 | 7 | 24 | 9 | 17 | 5 | 4 | 2 | 6 | 8 | 4 | 0 |
|  |  | Tu | 0 | 0 | 0 | 1 | 1 | 7 | 0 | 0 | 1 | 1 | 0 | 0 | 0 |
|  |  | LOT | 2 | 2 | 4 | 10 | 1 | 11 | 1 | 5 | 1 | 4 | 3 | 3 | 0 |
|  |  | OB | 0 | 0 | 0 | 1 | 0 | 0 | 0 | 0 | 0 | 1 | 0 | 0 | 0 |
| Amygdala | Cortical amygdala | AA | 1 | 4 | 6 | 25 | 3 | 24 | 2 | 6 | 4 | 12 | 3 | 3 | 0 |
|  |  | CxA | 4 | 2 | 15 | 33 | 10 | 26 | 5 | 12 | 8 | 4 | 10 | 3 | 1 |
|  |  | IPAC | 2 | 0 | 0 | 9 | 1 | 5 | 1 | 5 | 0 | 2 | 4 | 1 | 0 |
|  |  | ACo | 1 | 0 | 0 | 9 | 2 | 7 | 0 | 2 | 1 | 0 | 0 | 0 | 0 |
|  |  | PCo | 1 | 5 | 9 | 24 | 7 | 23 | 3 | 9 | 2 | 2 | 3 | 8 | 1 |
|  |  | APir | 2 | 3 | 14 | 30 | 12 | 15 | 2 | 9 | 2 | 3 | 2 | 12 | 1 |
|  | Basolateral complex | BLA | 7 | 6 | 3 | 133 | 11 | 57 | 4 | 6 | 2 | 16 | 4 | 21 | 3 |
|  |  | BLP | 5 | 2 | 2 | 22 | 6 | 9 | 3 | 2 | 0 | 1 | 0 | 2 | 1 |
|  |  | BLV | 0 | 0 | 0 | 0 | 0 | 2 | 0 | 0 | 0 | 0 | 1 | 0 | 0 |
|  |  | LA | 1 | 1 | 1 | 11 | 1 | 2 | 1 | 2 | 0 | 1 | 0 | 3 | 0 |
|  |  | BMA | 3 | 0 | 4 | 27 | 2 | 9 | 2 | 0 | 1 | 1 | 1 | 2 | 0 |
|  | Centromedial Amygdala | CeA | 0 | 0 | 1 | 0 | 0 | 1 | 0 | 2 | 1 | 0 | 0 | 0 | 0 |
|  |  | Med A | 2 | 0 | 5 | 40 | 3 | 29 | 5 | 4 | 4 | 4 | 1 | 7 | 0 |
| Brainstem | Brainstem | PAG | 0 | 0 | 0 | 3 | 0 | 5 | 3 | 1 | 0 | 0 | 1 | 0 | 0 |
|  |  | SN | 0 | 0 | 0 | 1 | 0 | 0 | 1 | 0 | 0 | 0 | 1 | 0 | 0 |
|  |  | VTA | 0 | 0 | 0 | 3 | 0 | 1 | 0 | 0 | 0 | 1 | 1 | 0 | 0 |
|  |  | Rn | 3 | 3 | 3 | 30 | 3 | 25 | 3 | 2 | 4 | 1 | 8 | 5 | 0 |
|  |  | Rt | 0 | 0 | 0 | 7 | 0 | 1 | 1 | 0 | 0 | 1 | 0 | 0 | 0 |
|  |  | Pn | 0 | 0 | 1 | 6 | 0 | 3 | 0 | 0 | 0 | 0 | 1 | 0 | 0 |
|  |  | OtherBS | 0 | 0 | 2 | 7 | 0 | 5 | 0 | 2 | 1 | 0 | 1 | 0 | 0 |
|  | | Total | 298 | 269 | 364 | 2458 | 454 | 1392 | 217 | 228 | 280 | 368 | 316 | 434 | 118 |

Table 7-2: Contralateral inputs in rabies tracing dataset

| Coarse categorizations | | Region | Contralateral input cells per region in animals Rb1-Rb13 | | | | | | | | | | | | |
| --- | --- | --- | --- | --- | --- | --- | --- | --- | --- | --- | --- | --- | --- | --- | --- |
|  |  |  | Rb1 | Rb2 | Rb3 | Rb4 | Rb5 | Rb6 | Rb7 | Rb8 | Rb9 | Rb10 | Rb11 | Rb12 | Rb13 |
| Cortical | Prefrontal cortex | rIns | 0 | 0 | 0 | 1 | 0 | 1 | 0 | 0 | 0 | 0 | 0 | 2 | 0 |
|  |  | LO | 0 | 0 | 0 | 4 | 1 | 4 | 0 | 0 | 0 | 0 | 1 | 0 | 0 |
|  |  | VLO | 0 | 1 | 1 | 15 | 2 | 4 | 1 | 0 | 0 | 0 | 1 | 3 | 0 |
|  |  | VO | 1 | 0 | 0 | 2 | 0 | 1 | 1 | 0 | 1 | 3 | 0 | 0 | 0 |
|  |  | MO | 1 | 0 | 1 | 11 | 2 | 2 | 1 | 1 | 0 | 0 | 0 | 4 | 1 |
|  |  | PL | 2 | 1 | 2 | 36 | 1 | 16 | 3 | 1 | 1 | 5 | 3 | 4 | 2 |
|  |  | IL | 2 | 0 | 2 | 9 | 4 | 2 | 1 | 0 | 1 | 0 | 1 | 0 | 0 |
|  |  | DPed | 0 | 0 | 0 | 1 | 0 | 0 | 0 | 0 | 0 | 0 | 0 | 0 | 0 |
|  | Cingulate cortex | dACC | 4 | 0 | 0 | 33 | 1 | 11 | 4 | 0 | 5 | 5 | 1 | 6 | 1 |
|  |  | vACC | 3 | 2 | 2 | 33 | 2 | 20 | 5 | 0 | 7 | 7 | 7 | 8 | 1 |
|  |  | RSp | 0 | 0 | 0 | 0 | 0 | 0 | 0 | 0 | 0 | 0 | 1 | 1 | 0 |
|  | Parahippocampal region | LEC | 0 | 0 | 1 | 2 | 2 | 4 | 0 | 0 | 1 | 0 | 0 | 0 | 0 |
|  |  | MEC* | 0 | 0 | 1 | 12 | 1 | 0 | 0 | 0 | 0 | 0 | 0 | 0 | 0 |
|  |  | PER | 0 | 0 | 1 | 1 | 0 | 2 | 0 | 0 | 0 | 1 | 0 | 0 | 0 |
|  |  | POR* | 0 | 0 | 0 | 1 | 0 | 0 | 0 | 0 | 0 | 0 | 0 | 0 | 0 |
|  | Hippocampal formation | CA1 | 0 | 0 | 0 | 0 | 0 | 0 | 1 | 0 | 0 | 0 | 0 | 0 | 0 |
|  |  | CA2 | 0 | 0 | 0 | 0 | 0 | 0 | 0 | 0 | 1 | 0 | 0 | 0 | 0 |
|  |  | CA3 | 0 | 0 | 0 | 12 | 0 | 0 | 0 | 0 | 0 | 0 | 0 | 0 | 0 |
|  |  | DG | 0 | 0 | 0 | 0 | 0 | 0 | 0 | 0 | 0 | 1 | 0 | 0 | 0 |
|  |  | Sub | 0 | 0 | 0 | 0 | 1 | 0 | 0 | 1 | 0 | 0 | 0 | 0 | 0 |
|  | Sensorimotor cortex | SSc | 0 | 0 | 1 | 6 | 0 | 3 | 2 | 0 | 0 | 0 | 0 | 1 | 0 |
|  |  | Aud c | 0 | 0 | 0 | 1 | 0 | 0 | 0 | 0 | 0 | 1 | 0 | 0 | 0 |
|  |  | Vis c | 0 | 0 | 0 | 0 | 0 | 1 | 0 | 0 | 0 | 1 | 0 | 0 | 0 |
|  |  | M1 | 0 | 1 | 0 | 14 | 0 | 1 | 0 | 0 | 2 | 0 | 0 | 1 | 1 |
|  |  | rM2 | 0 | 1 | 0 | 15 | 0 | 2 | 0 | 0 | 0 | 0 | 0 | 2 | 0 |
|  |  | cM2 | 2 | 0 | 0 | 12 | 1 | 5 | 2 | 1 | 2 | 2 | 1 | 3 | 0 |
|  | Other cortices | TeA | 0 | 0 | 1 | 0 | 0 | 0 | 0 | 0 | 0 | 0 | 0 | 0 | 0 |
|  |  | Par c | 0 | 0 | 0 | 1 | 0 | 0 | 0 | 0 | 0 | 0 | 0 | 0 | 0 |
| Subcortical | Basal forebrain | NAc | 0 | 0 | 0 | 3 | 0 | 0 | 0 | 0 | 0 | 1 | 1 | 0 | 0 |
|  |  | NDB | 1 | 1 | 0 | 1 | 0 | 2 | 0 | 0 | 0 | 1 | 0 | 1 | 0 |
|  | Thalamus | Th ANG | 0 | 0 | 0 | 0 | 0 | 0 | 0 | 0 | 0 | 0 | 0 | 1 | 0 |
|  |  | Th IL | 0 | 0 | 0 | 1 | 0 | 0 | 0 | 0 | 0 | 0 | 0 | 0 | 0 |
|  |  | Th MD | 0 | 0 | 0 | 1 | 0 | 0 | 0 | 1 | 0 | 0 | 0 | 0 | 0 |
|  |  | MetaTh | 0 | 0 | 0 | 0 | 0 | 0 | 0 | 1 | 0 | 0 | 0 | 0 | 0 |
|  | Hypothalamus | LH | 0 | 0 | 0 | 4 | 0 | 2 | 1 | 0 | 1 | 1 | 0 | 0 | 0 |
|  |  | MH | 0 | 0 | 0 | 0 | 0 | 0 | 0 | 0 | 0 | 0 | 0 | 1 | 0 |
|  | Other subcortical areas | ZI | 0 | 0 | 0 | 0 | 0 | 1 | 0 | 0 | 0 | 0 | 0 | 0 | 0 |
| Olfactory | Piriform area | rPir | 0 | 0 | 2 | 6 | 0 | 7 | 0 | 0 | 0 | 1 | 1 | 1 | 0 |
|  |  | cPir | 0 | 0 | 2 | 2 | 0 | 0 | 0 | 0 | 0 | 0 | 2 | 0 | 1 |
|  | Anterior olfactory area | AOL | 0 | 0 | 0 | 2 | 0 | 5 | 0 | 0 | 0 | 0 | 1 | 0 | 0 |
|  |  | AOM | 1 | 5 | 1 | 33 | 1 | 10 | 0 | 2 | 6 | 5 | 6 | 3 | 3 |
|  |  | AOV/P | 1 | 2 | 1 | 12 | 1 | 7 | 0 | 0 | 2 | 0 | 3 | 0 | 0 |
|  | Other olfactory areas | DTT | 0 | 0 | 0 | 0 | 0 | 0 | 0 | 0 | 0 | 1 | 1 | 0 | 0 |
|  |  | LOT | 1 | 0 | 0 | 2 | 0 | 2 | 0 | 0 | 0 | 2 | 1 | 0 | 0 |
| Amygdala | Cortical amygdala | AA | 1 | 1 | 0 | 2 | 0 | 0 | 0 | 1 | 0 | 0 | 0 | 0 | 0 |
|  |  | PCo | 0 | 0 | 1 | 0 | 0 | 0 | 0 | 0 | 0 | 0 | 0 | 0 | 0 |
|  | Basolateral complex | BLA | 2 | 0 | 0 | 29 | 2 | 7 | 1 | 2 | 2 | 4 | 0 | 1 | 0 |
|  |  | BLP | 1 | 1 | 0 | 2 | 0 | 0 | 0 | 0 | 0 | 0 | 0 | 0 | 0 |
|  |  | BLV | 0 | 0 | 0 | 0 | 0 | 1 | 0 | 0 | 0 | 0 | 0 | 0 | 0 |
|  |  | LA | 0 | 1 | 0 | 0 | 0 | 0 | 0 | 0 | 0 | 0 | 0 | 0 | 0 |
|  |  | BMA | 0 | 0 | 3 | 2 | 0 | 1 | 0 | 0 | 0 | 0 | 0 | 1 | 0 |
| BS | Brainstem | Rt | 0 | 0 | 0 | 2 | 0 | 0 | 0 | 0 | 0 | 1 | 0 | 0 | 0 |
|  |  | Pn | 0 | 0 | 0 | 2 | 0 | 1 | 0 | 0 | 0 | 0 | 1 | 0 | 0 |
|  |  | Sum | 23 | 17 | 23 | 328 | 22 | 125 | 23 | 11 | 32 | 43 | 33 | 44 | 10 |

* Inputs from MEC and POR could not be unequivocally classified as ipsi- or contralateral since the hemispheres were detached from the rest of the tissue in the caudal-most parts of the brain.
